# New Approaches to Identifying and Reducing the Global Burden of Disease From Pollution

**DOI:** 10.1029/2018GH000167

**Published:** 2020-03-25

**Authors:** Gabriel Filippelli, Susan Anenberg, Mark Taylor, Alexander van Geen, Haneen Khreis

**Affiliations:** ^1^ Department of Earth Sciences and Center for Urban Health Indiana University‐Purdue University at Indianapolis (IUPUI) Indianapolis IN USA; ^2^ Environmental Resilience Institute Indiana University Bloomington IN USA; ^3^ Milken Institute, School of Public Health George Washington University Washington DC USA; ^4^ Department of Environmental Sciences Macquarie University Sydney New South Wales Australia; ^5^ Lamont‐Doherty Earth Observatory Columbia University New York NY USA; ^6^ Texas A&M Transportation Institute Texas A&M University College Station TX USA

**Keywords:** pollution, lead, air quality, citizen science, soil, dust

## Abstract

Pollution from multiple sources causes significant disease and death worldwide. Some sources are legacy, such as heavy metals accumulated in soils, and some are current, such as particulate matter. Because the global burden of disease from pollution is so high, it is important to identify legacy and current sources and to develop and implement effective techniques to reduce human exposure. But many limitations exist in our understanding of the distribution and transport processes of pollutants themselves, as well as the complicated overprint of human behavior and susceptibility.

New approaches are being developed to identify and eliminate pollution in multiple environments. Community‐scale detection of geogenic arsenic and fluoride in Bangladesh is helping to map the distribution of these harmful elements in drinking water. Biosensors such as bees and their honey are being used to measure heavy metal contamination in cities such as Vancouver and Sydney. Drone‐based remote sensors are being used to map metal hot spots in soils from former mining regions in Zambia and Mozambique. The explosion of low‐cost air monitors has allowed researchers to build dense air quality sensing networks to capture ephemeral and local releases of harmful materials, building on other developments in personal exposure sensing. And citizen science is helping communities without adequate resources measure their own environments and in this way gain agency in controlling local pollution exposure sources and/or alerting authorities to environmental hazards. The future of GeoHealth will depend on building on these developments and others to protect a growing population from multiple pollution exposure risks.

## Key Points

- Legacy and current pollutants are a major global cause of disease and death
- Many detection and mitigation tools are not adequate for identifying pollution hot spots in communities, particularly vulnerable ones
- The next century of AGU will usher in novel, community‐focused techniques and approaches to mapping pollutants and reducing exposure

## 1 Introduction

Progress in tracking pollution from source to humans to disease has generally been singular in focus—one chemical, one pathway, and one human. As we will briefly outline here, 100 years of progress using this approach has yielded remarkable public health breakthroughs. These include steep reductions in sulfur emissions that previously killed thousands in various “fog” events in the 1900s, identification and mitigation of arsenic‐laden drinking water wells in Southeast Asia, and elimination of the toxic metal lead from most product streams (although much more about the unfinished story of lead to follow), for example. Together with significant advances in vaccine distribution and health access, these measures have arguably led to the healthiest human populace ever in terms of lifespan and infant survival (Myers et al., 2017). But the main focus of this analysis is not the past century but rather the next one.

The forward focus of this contribution is reinforced by a major Lancet Commission Report on the terrifying toll that pollution has on global morbidity and mortality (Landrigan et al., 2017). This report highlights global research showing that pollution alone kills 9 million people every year (Landrigan et al., 2017), 15 times more deaths than from all wars and violence combined. The quality of air, water, and land is diminishing in many parts of the world because of increasing global pollution (Landrigan et al., 2017; Shaffer et al., 2019), and this degradation is being borne disproportionately by poorer nations without adequate environmental protections in place for their pollutions (Myers et al., 2017). Much of this pollution has a long lifespan in the environment, and thus, if a pollution revolution occurs tomorrow, the legacy contaminants will remain in our environment for centuries (Filippelli, 2018).

The major advances in the coming century will come in mobilizing the science, the policy, and the action to confront these issues. It will involve engaging exposure science in a more convergent manner, engaging a broader range of expertise including those now often at the table—geoscientists and health scientists, managers, engineers, policy makers, and communities themselves. This is one of the reasons why the journal *GeoHealth* was born, and the AGU GeoHealth section was launched shortly thereafter, and it is thus fitting that this Centennial contribution appears here.

## 2 The Complicated Pathway From Pollution to Exposure to Disease

**Figure 1:**
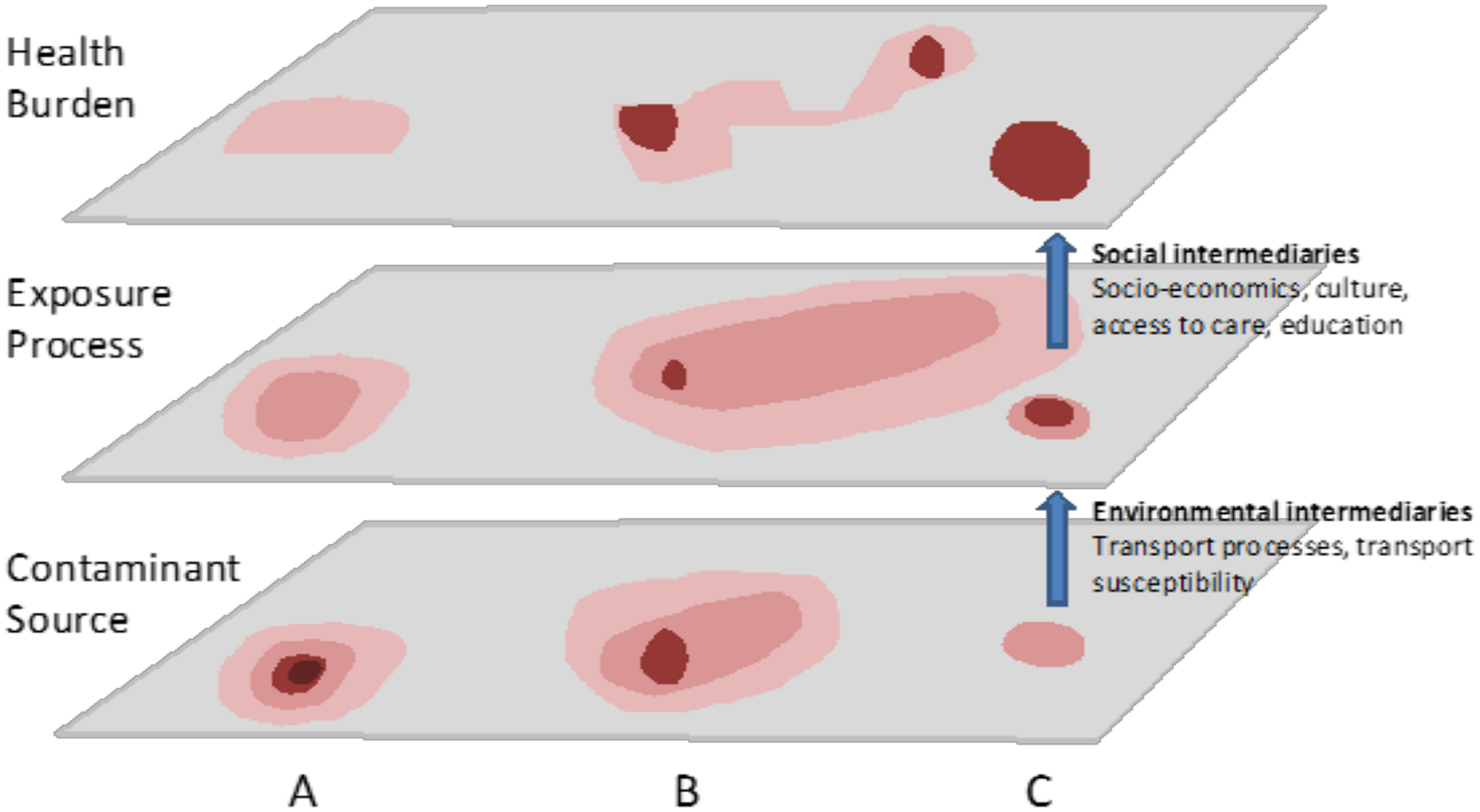
Schematic of pollution repositories, exposure pathways, and disparities in health outcomes. (a) Represents a contaminant (zinc) with limited toxicity, (b) a contaminant (lead) with significant community vulnerability patterns, and (c) a contaminant (mercury) with limited exposure pathways (fish) but strong toxicity for those exposed (from Filippelli et al., 2015).

When examining the links between pollution and human health, it is important to consider the exposure mechanism(s), the exposure source media, the exposure vulnerability of communities, and the toxicant of health concern. The complication of this multilayer matrix is in and of itself one of the reasons why it is so difficult and can take so long to truly understand connections between exposure and disease (Figure 1). Each “layer” is governed by unique dynamics, often led by uniquely skilled investigators, and are disciplines or services with unique criteria for sharing data and for disseminating it. The case of the Flint, Michigan, water crisis provides an example of the failure to adequately integrate these sectors. Water managers switched supply sources to save money, the involved water engineers inadequately modeled the impacts of water chemistry on internal pipe deposit mineralogy, the surveillance system measured water chemistry from the finished water side of the system but not at the tap, and the public health system had to wait for hundreds of lead poisoned children showing up in clinics before the problem was traced back to its source, over a year later (Hanna‐Attisha et al., 2016; Sadler et al., 2017). Even once the water system is ultimately repaired, Flint, like many other older cities in the U.S., suffers from soil lead contamination, which remains unaddressed (e.g., Filippelli et al., 2015)—in Flint, it is likely responsible for ~50% of the peak blood lead levels even at the peak of the water crisis (Laidlaw et al., 2017). The links between lead poisoning, criminal misconduct, and the environmental injustice of this situation have been highlighted (e.g., Needleman et al., 2002), but it is not at all unique, and until we more fully integrate the geo‐side and health‐side in the area of pollution exposure and disease, we are bound to continue failing the health of people, and often the most vulnerable people.

The growth of data sets available that are geolocated has been a boon for researchers looking to integrate appropriate data “layers” spatially and temporally. In terms of pollution‐health studies, the spatial inventories of landfills, industrial facilities, roads, abandoned properties, and other potential sources of pollutants are often available through public records, including those available in the U.S. at the Environmental Protection Agency but also at numerous state and county level sources. Additionally, the growth of citizen‐science networks of instruments is another way that geolocated environmental data are now open to scientific research and discovery.

A limitation to integrating data layers that are geolocated comes largely in the human factor, where many countries, including the United States, have strong restrictions on the acquisition and analysis of human health data that could be identified at the individual level, for the very valid reason of protecting privacy. Various avenues do exist to acquire individual health data for analysis, largely through oversight by university and independent review boards, but even here, researchers can be hampered by the lack of central repositories of health data. Much as emission source data does not necessarily inform researchers about the spatial distribution of the deposition of pollutants, human health data are typically pinned to home location, or location of treating hospital or clinic. This location data do not take into the movement of the people whose data are being acquired, nor in the length of time they may stay at a particular location.

Even with these limitations of access and availability, great strides have been made in the past decade to integrate environmental and health data sets in useful ways. Examples of this integration include (1) detailed spatial and temporal analysis of urban temperatures and heat‐related morbidity (disease) and mortality (death; Johnson & Wilson, 2009), (2) analysis of the temporal correlation between the application of nitrate fertilizers and atrazine (a pesticide banned in Europe but widely used in the United States) and birth defects tied to early in utero exposure (Winchester et al. ,2009), and (3) the temporal relationship between atmospheric lead loading and levels of lead in children's blood (Laidlaw et al., 2012). For this reason, new research advances in pollution and health will largely require coordination and collaboration between researchers and research groups with expertise in environmental chemistry, transport, and fate analysis, geostatistics and biostatistics, disease epidemiology, sociology, geographic information systems, and medicine.

This Centennial contribution utilizes an “environmental sector” approach to understanding the past, present, and future of pollutants and human health. We focus on soil (and dust generated from soil). These are disciplinary distinctions in environmental science and in environmental regulation, but like all categorization schemes, this approach can suffer a bit from siloism. At a minimum, however, it provides a useful construct for understanding environmental exposure from multiple sources and is a tool for mapping out the past century of the science, and in helping to identify future developments and research needs.

Much of the soil pollution section will use the metal lead as an example of pollution exposure risks, in general, both because of the profound human health impact of global lead pollution (e.g., Landrigan et al., 2017) and because it exemplifies the challenges, and opportunities, of mitigation and ultimately of human health improvements. It is important to recognize that many pollution exposure issues involve simply poor education and/or few choices—the poisoning of millions by arsenic and fluoride in water in Southeast Asia is largely tied to the delayed nature of exposure‐disease manifestation plus the necessity for free and available water (Case Study 1). We will illuminate several of these future directions with “case studies” of novel approaches to identifying and eliminating pollution and improving global health.

**Case Study 1 Geogenic Toxicants in Groundwater**

Entirely natural processes can also lead to the heterogeneous distribution of a human health risk. High levels of arsenic (As) and fluoride (F) in well water are two examples whose severity and global impact have been well documented (Amini, Abbaspour, et al., 2008; Amini, Mueller, et al., 2008). What is perhaps less widely known is the extent to which spatial heterogeneity complicates prediction but also creates opportunities for rapid mitigation at very little if any monetary cost. This is important because chronic exposure to As from drinking well water has been linked to spontaneous abortions, stillbirths and infant mortality, inhibited intellectual and motor functions in children, and adult mortality from cardiovascular and other diseases (Flanagan et al., 2012; Quansah et al., 2015; Smith et al., 2000; Wasserman et al., 2016).

**Figure 2:**
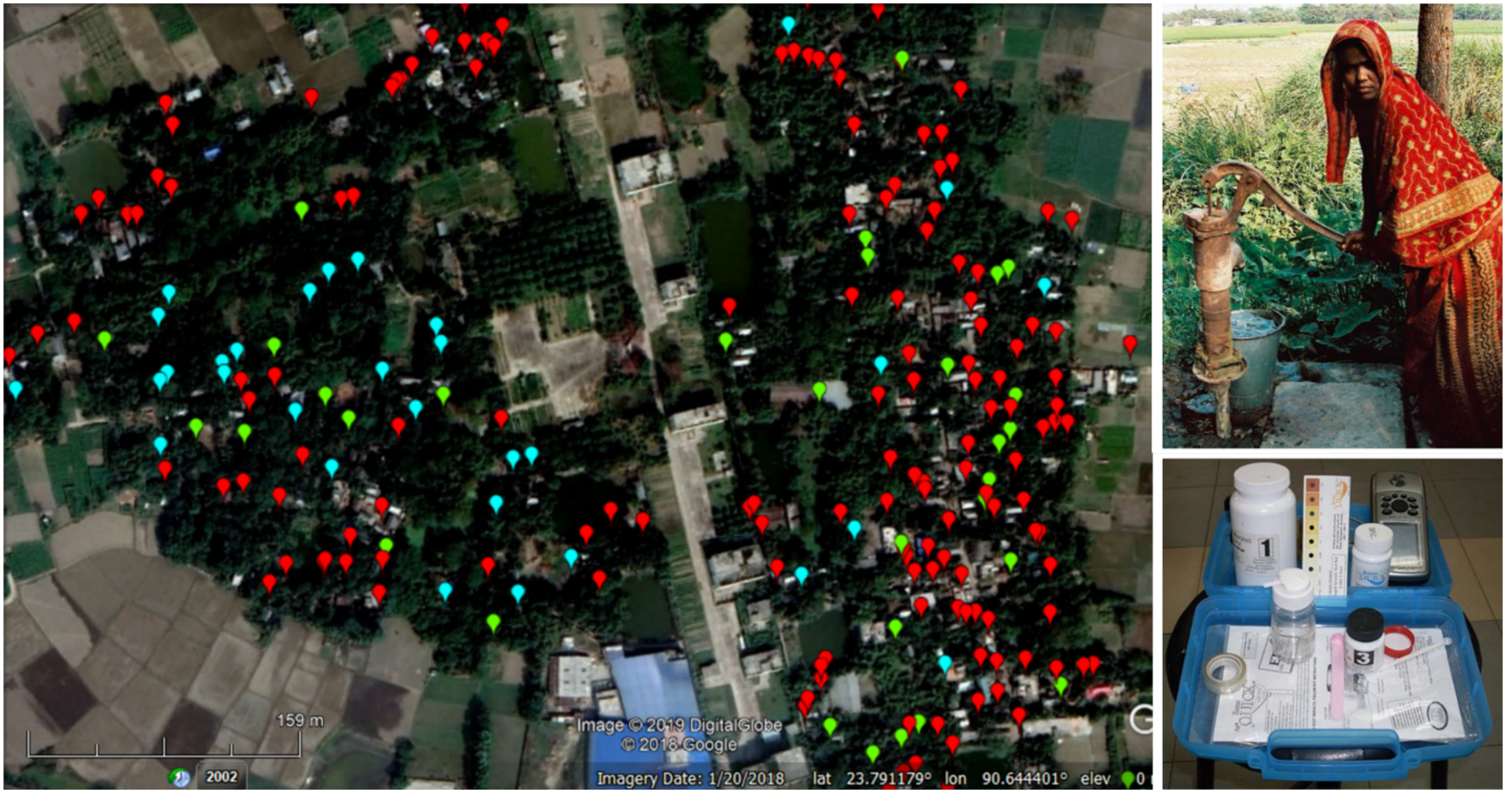
Distribution of As in hand‐pumped well water of a representative village of Araihazar upazila (subdistrict) of Bangladesh (van Geen et al., 2014). The survey was conducted in 2012–2013 by 10 local women equipped with the ITS EconoQuick kit (George et al., 2012). Symbols are color coded according to visual readings of the test strip of 0 and 10 (light blue), 25 and 50 (green), and 50–500 ppb (red—levels that are unsafe for drinking water).

The satellite image below (Figure 2) shows a typical village in Bangladesh, the country most affected by elevated levels of As in groundwater (BGS/DPHE, 2001). Also shown is the location and status with respect to As of dozens of handpumps installed by individual households. The distribution of As in well water shows that portions of the village are more affected but also that many of the less fortunate households live with walking distance of neighboring wells that is low in As. On the basis of blanket testing of all 6,000 wells within a larger 25 km^2^ area, van Geen et al. (2002) calculated that although half the wells were high in As, 90% of these wells were located within 100 m of low‐As well.

Over time, the limited number of medium‐ to long‐term studies that have been conducted have shown that when As concentration in well water are low, if they change at all, it will usually be only gradually (BGS/DPHE, 2001; McArthur et al., 2010). One of the reasons is that aquifer sediments, iron oxides, in particular, represent a much larger pool of As than what is contained in groundwater. Given that the two pools are to first order in equilibrium with respect to adsorptive exchange, this means that any perturbation in groundwater flow, for instance, has to modify the large exchangeable in aquifer sediment before it can measurably change As concentrations in groundwater (Fendorf et al., 2010). This type of buffering against changes in groundwater flow, also referred to as retardation, is important because it means that any arrangements needed to share the subset of safe wells in particular village are well worth making as a short‐term and possibly long‐term solution for reducing As exposure.

Spatial heterogeneity and temporal stability mean that from a public health and Earth science issue two decades ago, the problem of As in groundwater has evolved to one of access to information and incentives for sharing safe wells. There are now an estimated 10 million wells in Bangladesh, most of which have never been tested (Jamil et al., 2019). Testing in the laboratory of a such a large number of wells, which are replaced on average once a decade, is not realistic. There are several field kits on the market now that are entirely suitable for knowing whether a well contains 1, 10, 100, or even 1,000 ppb As (George et al., 2012). Households are willing to make an ~$100 investment to access water that is seemingly safe but are not willing to pay a few dollars for a test (Barnwal et al., 2017). This means that a massive, permanent testing program should be supported by the government. Beyond providing the information that is essential for well sharing in the short to medium term, the same test data can be the basis for targeting safe aquifers by individual households and the government (Jamil et al., 2019). For both the collection of test data and dissemination of test results, the growing use of smartphones in Bangladesh could improve this problem in the next few years.

## 3 Current and Legacy Pollution Sources From Soils

### 3.1 Soils and Soil‐Sourced Dust as a Human Health Concern

Soil is the geologic envelope of the Earth's surface that humans most interact with, whereas dust is a generic term, which can include the fraction of dust that is transported by airborne processes but can also include fine particles from a variety of anthropogenic practices. Here we restrict dust to the net sum of atmospherically deposited material leaving the actual suspended particulate matter for the following sections.

Soils develop via biogeochemical processes that occur over millennia (see Huggett, 1998, for a critical review). Soils support plants and crops and the vast majority of our terrestrial biosphere mass. As such, they contain contaminants that are geogenic in origin in addition to those of an anthropogenic nature (e.g., As in groundwater from the underlying geology in Bangladesh). Soils are the primary source of geogenic airborne dust. But soils are also susceptible to a host of human activities, including erosion and soil loss and contamination from a number of human‐applied chemicals (i.e., fertilizers, herbicides, and pesticides) and human‐emitted pollutants (i.e., lead, mercury, and organic chemicals). The hydrologic and geochemical absorptive capacity of clays and organic matter that dominate surface soils can result in the hyperaccumulation of some of these contaminants over time to levels that are dangerous for human exposure, both from the soil and from the dust generated by that soil. Furthermore, microbial interactions in soils and sediments can also convert relatively innocuous elements into lethal ones, such as the microbial methylation of mercury, which then enables it to enter the food web and be bioconcentrated to harmful levels in fish.

The terms “toxic” and “soil” and “human” just began creeping into the scientific literature soon after the birth of the American Geophysical Union, with the earliest clear link between soil chemistry and human health, based on a Google Scholar search, published in 1935 (Beath et al., 1935). In the Journal of Chronic Diseases, Schroeder and others (e.g., Schroeder et al., 1961) published a series of papers in the 1960s with the title “Abnormal trace metals in man: XYZ” surveying the periodic table and summarizing the state of the literature at that time on metal excess and human health. Interestingly, this was paired with a series of articles variously titled “Essential trace metals in man: XYZ” that tracked metal limitation and human health. These studies set the stage for many studies over the next half century on the exposure mechanisms (air, water, and soil/dust) and health impacts of metals. Since then, thousands of papers and several books have been published on the topic, and we have gained some understanding of the distribution, chemistry, toxicity, and exposure mechanisms of soils and dust to humans. In the following we discuss broadly the modern “state of science” for soil and dust pollution and health and focus on several examples of these mechanisms by focusing on several of the most widespread and harmful soil pollutants globally.

### 3.2 Soil Geochemistry and Human Exposure Potential

In these past decades, geochemists have been developing and employing a set of techniques to differentiate the mineral reactivity, or bioreactivity, of metals in a number of media, including soils, water, gastric fluid, and pulmonary fluid (Agemian & Chau, 1976; Sheppard & Thibault, 1992; Ure et al., 1992; Ruby et al., 1996; Hamel et al., 1998; Dollar et al., 2001; Plumlee et al., 2006; Reeder et al., 2006; Plumlee & Morman, 2011; Xie et al., 2012; Saunier et al., 2013), as it pertains to human health. Much of this work involves selective extractions in the lab and/or field‐based studies, using geochemical sensors, phytological responses, or animal models. Often these are using mineral reactivity to standardized extractants that mimic either natural fluids or biofluids (e.g., Tack & Verloo, 1995; Hamel et al., 1998; Ruby et al., 1996; Skowronski et al., 2010). Additionally, isotopic tracers have been used to identify mineral and metal transformations and/or biological uptake (e.g., Maddaloni et al., 1998; Guelke & von Blanckenburg, 2007). From these experiments, a set of reactivity terms has been defined and debated. These include (1) bioaccessibility, which is the elemental component that is released into solution from soil under a chemical digestion, and (2) bioavailable, which is that portion of the bioaccessible component that can be absorbed in the body via the gastrointestinal tract, the pulmonary system, and the skin (e.g., Alexander, 1982; Ruby et al., 1996; Herrchen et al., 1997; Ehlers & Luthy, 2003; Peijnenburg et al., 2007; Hedberg et al., 2010; Luo et al., 2012; Kumpiene et al., 2017). To note just one example of this distinction, in mining‐contaminated soils in Spain, Martinez‐Sanchez et al. (2012) found that whereas total arsenic was dangerously high (well over the 15 ppm soil exposure guidelines for a child set by the ATSDR, 2007), the bioaccessibility of that arsenic was relatively low, and thus, also, the bioavailability was low.

Direct ingestion of soil or pollutant particles within the soil matrix is a common exposure pathway for metals (or inhalation, mucus trapping, and swallowing, which has effectively the same net result; U.S. EPA, 2007). The human gastric system is aggressive, and the subtle accessibility experiments appropriate for ecosystems with a relatively small range of pH are not good models for human uptake. The stomach has a pH of 1.5–3.5, well below that of all normal surface environments, and the intestines have a pH of 6 to 7.4 (variation largely driven by stage within the intestinal tract) and myriad digestive enzymes and microbiota. This large sequential range in pH and the very matrix heavy nature of the gastric system has made moot many of the other ecological digestion techniques used and thus has been more effectively modeled by simulated gastric digestions in lab‐based studies.

Capitalizing on the fact that pollutant concentration might not be as important as uptake potential (i.e., the bioavailable fraction of the total), a number of remediation technologies have been evolving to immobilize harmful pollutants in place in cases where mitigation by removal of soil is not feasible or economical. Clay minerals have been used in several field studies for cadmium (Cd) and lead (Pb) remediation of contaminated soils and sediments (e.g., Sun et al., 2015). But perhaps the most effective in situ remediation approach for metals has been mineralization capture with phosphate minerals (Miretzky & Fernandez‐Cirelli, 2008; Zia et al., 2011). After various laboratory‐based experiments have shown that the addition of large amounts phosphate into soils contaminated by lead and other metals significantly lowers their bioavailability through formation of a metal‐phosphate mineral, the U.S. EPA has funded several large‐scale field applications of phosphate in lead‐contaminated communities (e.g., Freeman, 2012; U.S. EPA, 2011). The phosphate was applied in the form of crushed fish bones, which is composed almost entirely of hydroxyapatite. The advantages of this approach are (1) the beneficial utilization of a material, fish bones, that would otherwise be a waste product of fish packing, (2) the in‐site immobilization of lead without the need for disruptive, property‐by‐property soil removal, and (3) the fertilization effect of the phosphate itself that is also released during bone decomposition. There is a potential for inadvertent remobilization of Pb through changing the soil pH. Additionally, the mineral formation kinetics are slow, and thus, the treated properties have to have proper irrigation and soil stabilization, typically achieved by adding a layer of topsoil over the fish bone‐treated layer. This latter step alone has proven successful in several studies (e.g., Filippelli et al., 2015; Mielke et al., 2011), and unlike the slow process of fish bone remineralization, the improvement is immediate.

### 3.3 Spatial Distribution of Soil Pollution

**Figure 3:**
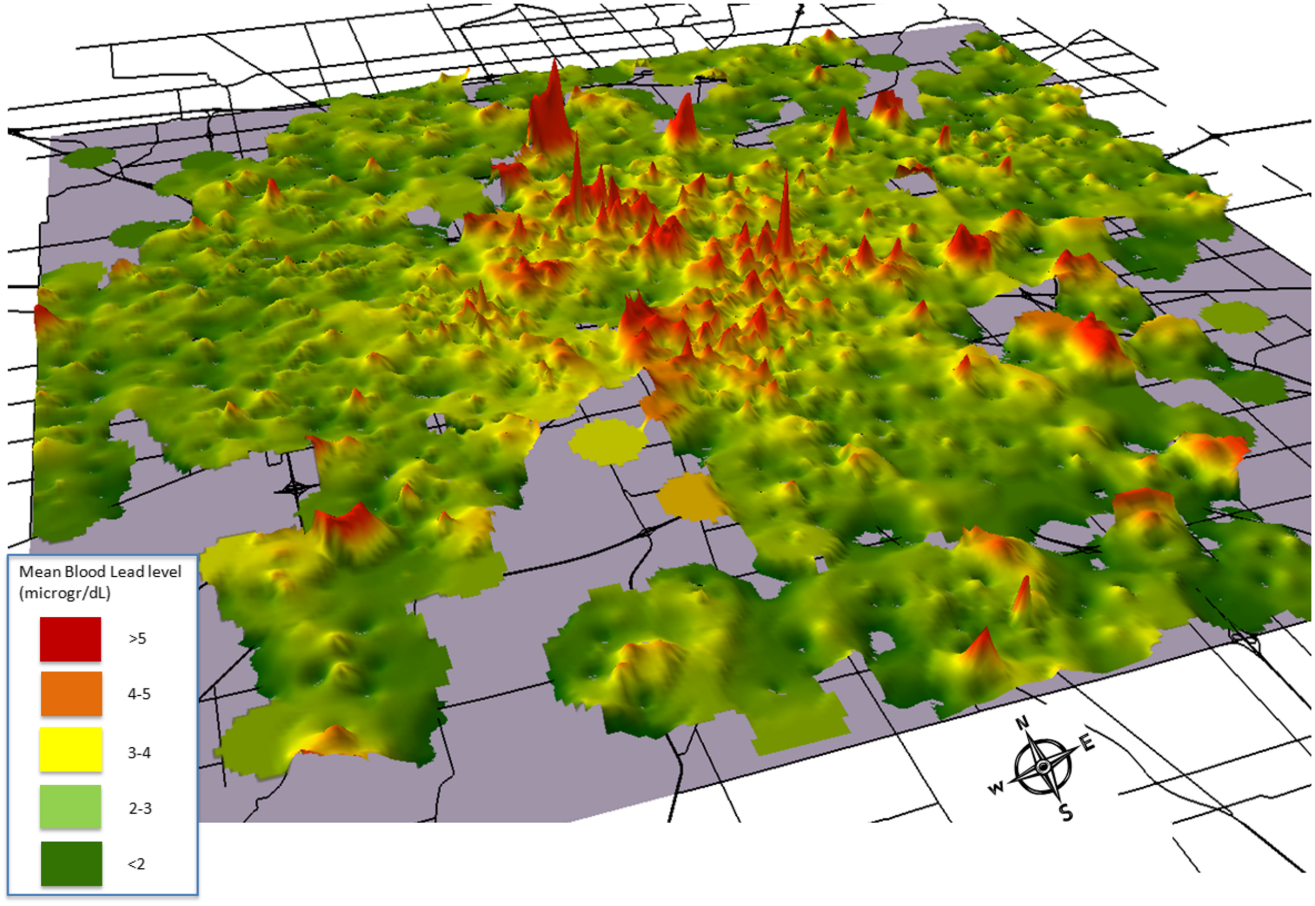
Blood lead levels of children in Indianapolis, Indiana, USA, for the period February 2002 to December 2008 (*n* = 12,431) for children between the ages of 0 and 5.99 years old in Indianapolis, Indiana (area = 1,044 km^2^; from Filippelli et al., 2012).

Soils bear a legacy of hundreds to thousands of years of human occupation and industrialization (e.g., Bellinger, 2011; Chambers et al., 2016; Filippelli & Taylor, 2018). Some pollutants, including many metals with poor mobility in soil media (i.e., lead, cadmium, and copper), have soil residence times on the order of hundreds to thousands of years. Additionally, if the major pathway was via airborne deposition, surface soils have concentrated decades to centuries of deposition of these low‐mobility metals right near the surface. The problem is that soil metal concentrations are highly heterogeneous at the small scale because of multiple sources (house paint, automobile exhaust and debris, particulate matter from utilities, industrial sources, etc.), and barring the identification of particular emission sources and sinks, these metal hot spots prove exceedingly difficult to pinpoint (Filippelli et al., 2015; Laidlaw et al., 2017; Mielke et al., 2019; Zahran et al., 2013). Indeed, we often need to resort to spatial distribution of metal‐poisoning children to map back to potential sources (Figure 3).

We need a better understanding of the soil metal dynamics and patterns of distribution and associations in the urban environment, with an eye toward identifying those processes and sources that have the highest potential to cause human harm and ultimately remediating those sources in a surgical fashion (Case Study 2).

**Case Study 2 Pollution Biosensing**

Understanding, measuring, assessing, and responding to environmental contaminant risk is an ever‐increasing requirement for industry, government, and individuals. Much of industry and government environmental monitoring is completed using standard methods and approaches (e.g., in Australia such as those promulgated by Australian Standards). However, the wider community now has unprecedent to environmental data and multiple sensing tools to better understand their environment (https://www.liebertpub.com/doi/10.1089/env.2016.0044., Kaufman et al., 2017).

Rising out of the need to develop new and innovative approaches to assessing temporal and spatial shifts in contaminants has been the use of terrestrial biomarkers, which are relatively easy to collect and analyze but provide dense data. Examples that people can readily connect environment to contamination include the use of historic lichens and fungi (e.g., Flegal et al., 2010; Wu et al., 2017), decadal records of stored red wine (Kristensen et al., 2016; Medina et al., 2000), and bees and honey (Zhou et al., 2017; Zhou et al., 2018; Smith et al., 2019).

These studies show unequivocally that environmental contamination since the industrial revolution has grossly contamination urban environments (Kristensen et al., 2017), with distal oceans and ice masses being affected but to a much lesser extent (McConnell et al., 2014; More et al., 2017; Ndungu et al., 2016). The data all show that environmental contamination from mining and smelting of lead and the use in leaded gasoline have resulted in clear shifts in lead concentrations and isotopic compositions over time. In particular, there were changes in lead concentrations and isotopic compositions associated with the start of leaded gasoline use in the early twentieth century followed by cessation of use across most of the global by the turn of the next century.

**Figure 4:**
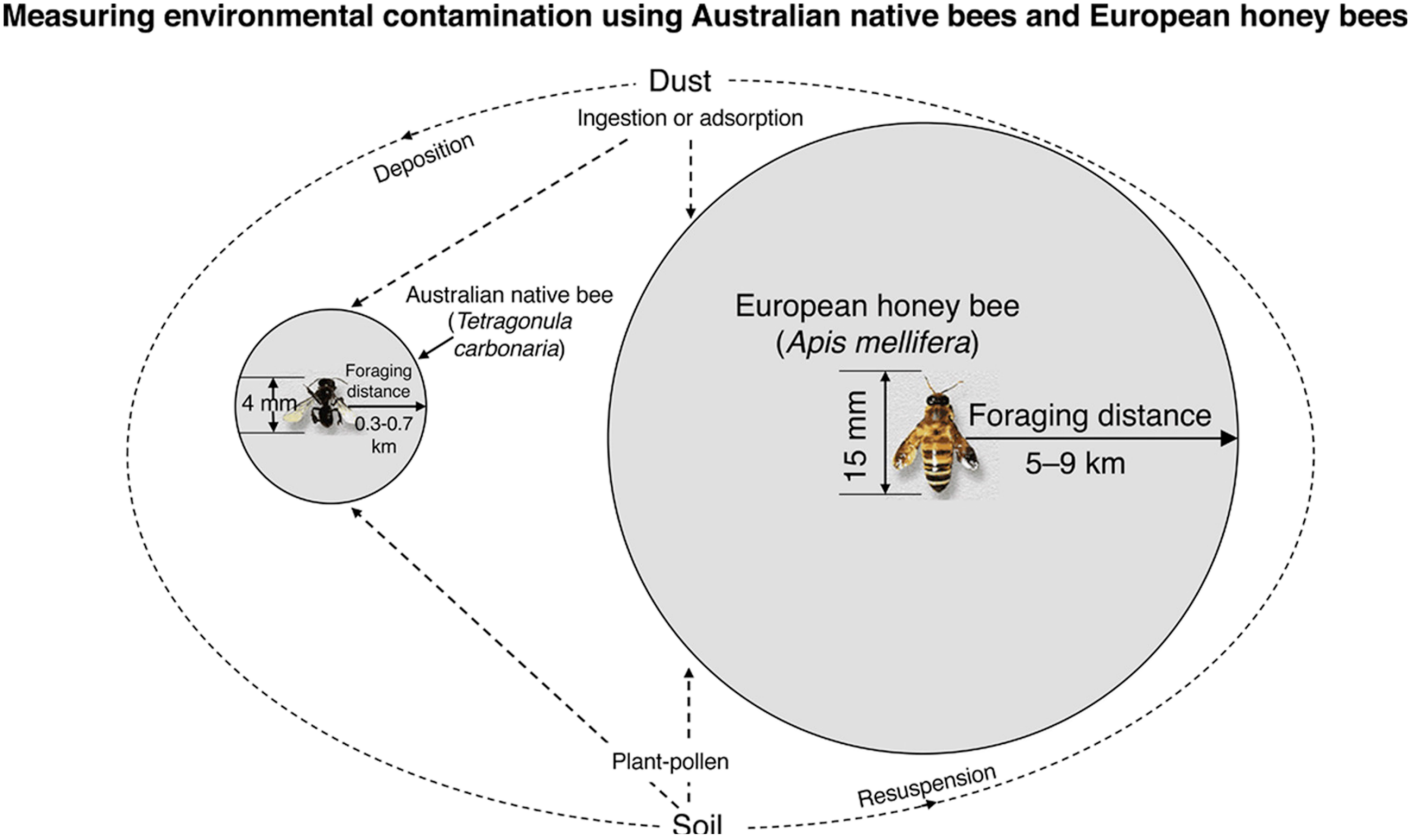
Foraging distance and organism size of Australian native versus imported European bee populations (from Zhou et al., 2018).

Recent studies out of Australia (Zhou et al., 2018) and Canada (Smith et al., 2019) using a variety of detailed spatial sampling and geochemical analyses of environmental media (soils and dusts) and bees honey and wax have confirmed that both native species and the more common *Apis Mellifera* (European honey bees) are excellent trace element biomarkers for inorganic trace metal contaminants (Figure 4; Negri et al., 2015; Van Der Steen et al., 2015; Taylor, 2019). These studies are important because bees and honey production is growing rapidly in popularity in urban environments, producing thousands of opportunities to understand the prevalence, distribution, and impact of the recycling of potential toxic contaminants into food and ecological systems. The Canadian study of bees and honey in Vancouver compared trace elemental concentrations to those found in local dust and air samples and proxies such as lichens and tree rings. Their analyses showed different environmental and biomarkers contained corresponding data, providing additional evidence for the reliability of bees and honey as biomarkers for anthropogenic contamination.

Even though global populations are becoming more urbanized and are consuming more, there is an unprecedented awareness of human impact on the environment (EEA, 2016) with related concerns about the quality and source of the food (e.g., Zhou, Taylor, Davies, & Prasad, 2018) produced in potentially contaminated landscapes (U.S. EPA, 2014). Almost paradoxically, in the center of contamination—our global cities—urban gardening has risen to an all‐time high, with ~35% of Americans (NGA, 2014) and 52% of Australians producing some food from their garden space (TAI, 2014).

**Figure 5:**
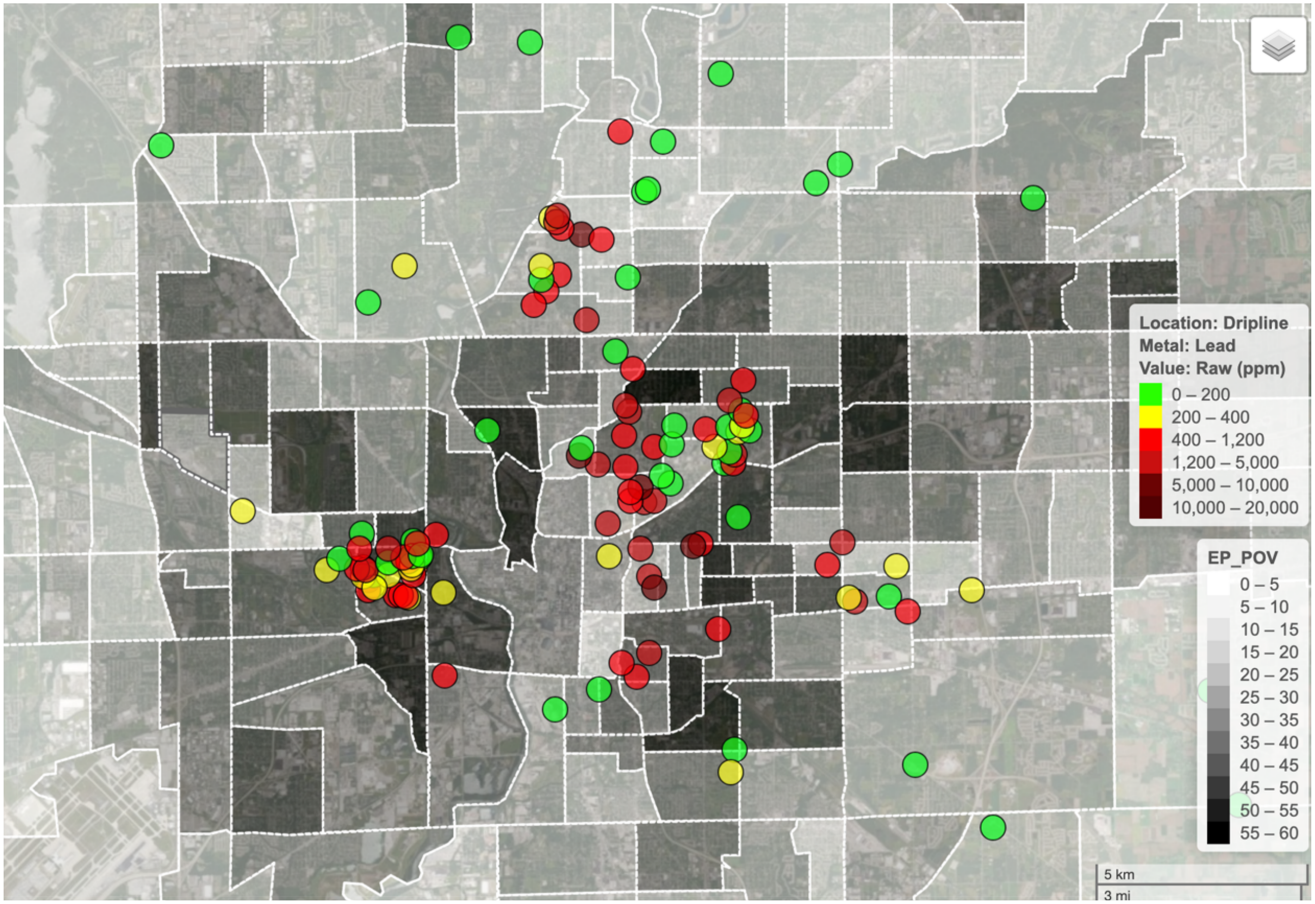
Soil lead concentrations at the home dripline from Indianapolis, Indiana (USA) in colored circles, and percent of population in poverty at the census block level (http://www.mapmyenvironment.com/).

Soils are an enormous reservoir of lithogenic and atmospheric trace elements, some of which have been significantly adulterated by anthropogenic emissions and depositions, in particular lead for paints and leaded gasoline emissions (Ashrafzadeh et al., 2018; Filippelli & Taylor, 2018; Rouillon et al., 2017). Given that communities are so heavily invested in their own welfare and producing clean and safe food from a reliable source, they have proven to be an incredible resource for the biosensing of soils for urban trace metal contaminants. The citizen science soil sampling programs run out of Indiana University‐Purdue University Indianapolis (USA) and Macquarie University (Sydney, Australia) have resulted in >15,000 soils from >3,300 homes being tested for a range of known metal contaminants by XRF (Figure 5). Participants are provided with a results report containing information about soil guidelines and additional resources that contain advice on soil contamination and prevention tips to reduce exposures (https://research.science.mq.edu.au/vegesafe/). These programs have assisted urban gardeners by providing them with knowledge of their own environment and ways to reduce exposure.

To classify legacy soil pollution risk, some generalities based on the location and land use are useful. Here, we break this up into urban settings and locations near mining facilities or abandoned mines. One priority is understanding and mitigating those cases where pollutant levels are high and/or exposure potential is high (e.g., arid and/or windy environments), and areas with a high exposure impact, largely relating to population size. It is important to note that other human‐impacted environments exist, such as agricultural areas, military facilities, and specialty chemical facilities, each with their own unique exposure risks.

#### 3.3.1 Urban Patterns

##### 3.3.1.1 Roadways

Roads are linear point sources of pollution, and a strong relationship exists between morbidity/mortality and distance that a person lives from a road. But this generalization masks a variety of individual pollutant and resultant exposure dynamics that are important to untangle. First, it is not just proximity but also traffic density that matters. Additionally, roadways create eddy systems that transport those solid‐phase contaminants some distance from the roadway itself, and thus, traffic speed and road edge design (barrier, no barrier) make a difference. Second, vehicles emit a vast and varying array of pollutants through their tailpipes, their undercarriage, and their tires and brakes. These tailpipe pollutants include particulate matter and gases from primary and secondary products of fossil fuel combustion (more on these in the section 0.3). This emission profile varies substantially by fuel type, fuel quality, age, and technology of emission control systems, and in the case of leaded gasoline, fuel supplements. Finally, vehicle components themselves are slowly but surely degrading and leaving residues of this process behind on the road, including various plating materials high in chromium, significant amounts of zinc from tire wear, copper and copper alloys from brake wear, and lead wheel weights that can fall from wheels and be degraded along roadways.

**Figure 6:**
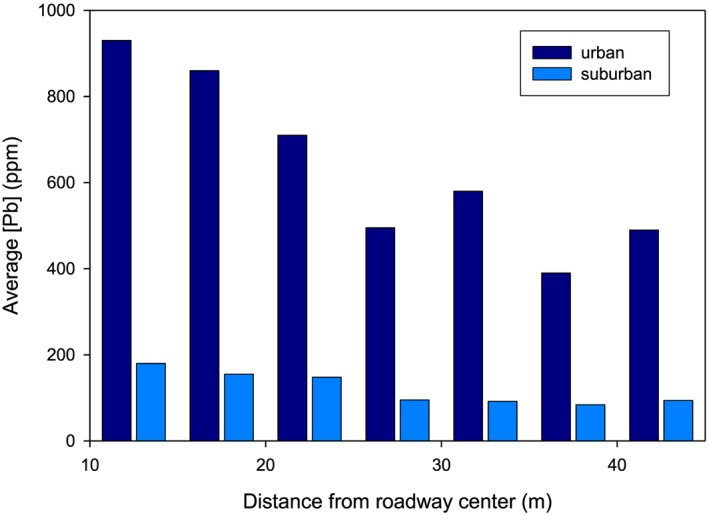
Average lead concentrations in surface soil as a function of distance from the roadway in urban and suburban transects, revealing the legacy signature of lead deposition from burning leaded gasoline many decades earlier (from Filippelli et al., 2005).

Cities, with their typical high density of roadways, large traffic volumes, and historical records of leaded gasoline consumption, typically contain a crisscross of soil contamination that might have been originally restricted to near the roadway but are now more diffuse due to soil and dust mobilization. One example of this roadway legacy is seen in lead, which was emitted from tailpipes for decades with the combustion of leaded gasoline. Lead was almost completely phased out of gasoline by 1980 in the U.S., but the legacy of this past use is still painted onto urban soils, with old major roadways showing a clear pattern of enrichment proximal to the road and gradual decrease by 30–50 m of the roadway edge to whatever the urban background is in that region (e.g., Filippelli et al., 2005; Figure 6). Interestingly, newer urban throughways, even though high density, only show a hint of this roadway effect, presumably because they were mostly used after leaded gasoline had been phased out.

##### 3.3.1.2 Housing

Lead‐based paint is a critical component of the urban lead load, as this was used for centuries before being phased out during the twentieth century. Much of this lead is still on older housing stock but is anything but immobile, as the degrading paint deposits both indoors and outdoors near the house exterior, where values can be several times higher than the average for the property as a whole (Filippelli et al., 2018). Other issues, including zinc, can also be elevated at the house dripline, either due to inclusion in paint and/or to the airborne fallout that occurs at the home barrier (Filippelli et al., 2018). Houses in urban (and indeed rural) environment can also contain myriad indoor issues, including a host of human‐produced chemicals (PFOAs, VOCs, and others; Filippelli, 2019), the toxicology of which is poorly constrained.

##### 3.3.1.3 Industrial Sources

Industrial facilities, particularly when colocated in urban areas as many have been for proximity to employees and distribution hubs, can pose serious health impacts in urban communities. Off‐site emissions or migration via dust resuspension can impact nearby communities (Brandon, 2013; Filippelli & Taylor, 2018). Indeed, even with emission controls increasingly implemented in many countries over the past 50+ years, modern facilities still emit a host of pollutants, including particulate matter (with attendant chemical pollutant loads) and volatile components that lead to poor air quality (e.g., EPA Emissions Inventories, 2019). In countries without adequate protections, these emissions can cripple communities with life‐shortening pollution (Dowling et al., 2016; Ericson et al., 2013). Those industries that are now well controlled can also have a legacy of past preregulatory emissions, making the identification and mitigation of these legacy impacts a critical issue. More concerning, however, are so‐called “Ghost Factories,” those facilities that emitted pollutants before modern regulations but which are now either abandoned or completely demolished. Often, inadequate records or site historical documentation is available to map out the potential past emissions from these facilities, making the identification and mitigation that much more challenging (USA Today, 2012).

#### 3.3.2 Mining Facilities

Mining and mineral extraction, as well as secondary processing of materials, concentrate potentially harmful minerals and elements and, in so doing, can pose risks from direct occupational exposures and indirect risks to families of workers and nearby localities if not tightly controlled. The growth of secondary mining and processing from nonprofessional recyclers has caused widespread contamination and poisoning of processers and indeed entire neighborhoods (Bose‐O'Reilly et al., 2017). Primary exposures to workers can be controlled and reduced by regulatory oversight and the use of Personal Protective Equipment (PPE), but one or both of these measures can fail.

**Figure 7:**
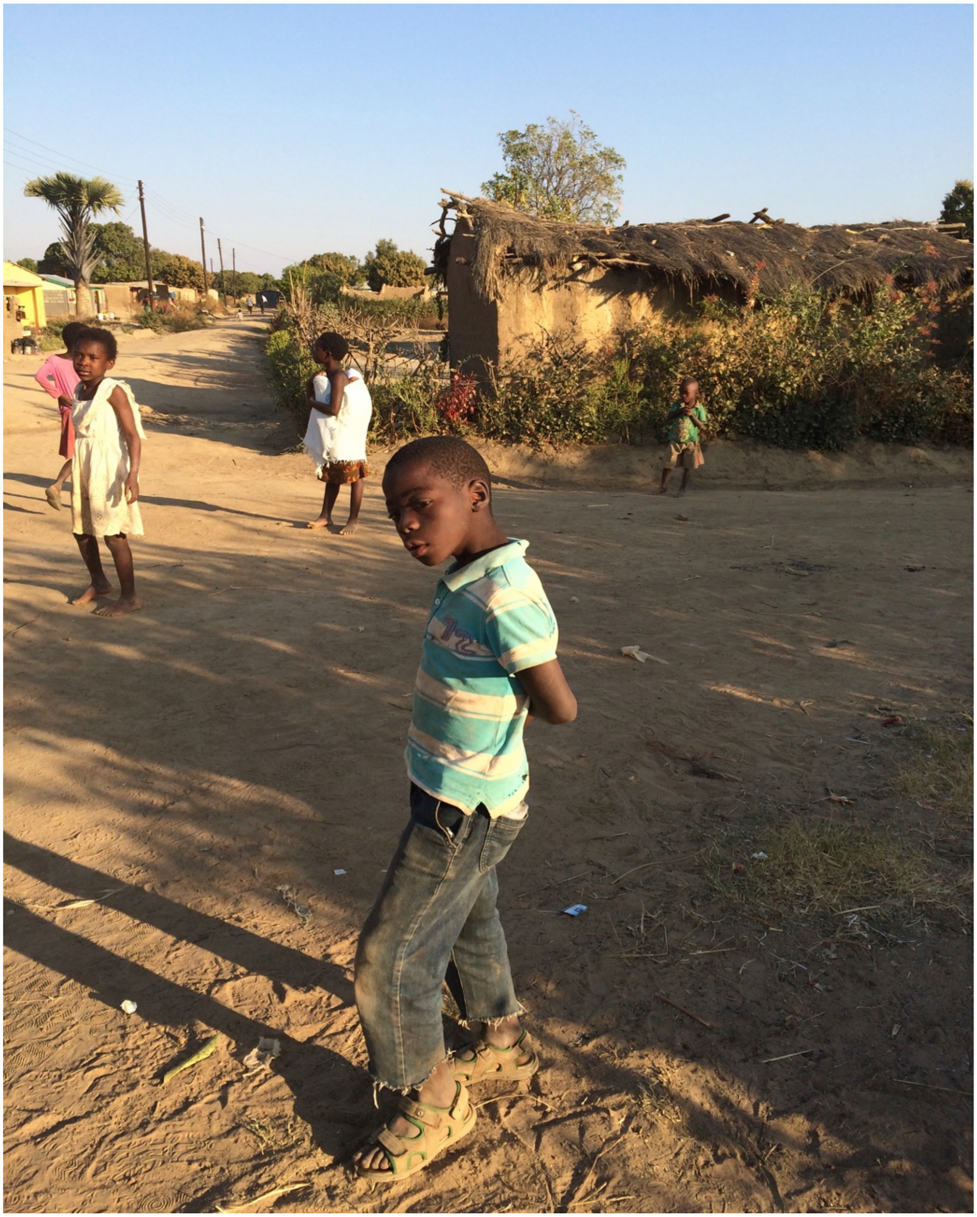
Over 95% of children living in the most affected townships of Kabwe, Zambia, had high blood lead levels over the intervention level set by many health agencies (Bose‐O'Reilly et al., 2017; photo taken by G. Filippelli).

Arguably more elusive is control of pollution in abandoned mining facilities and from secondary processing. One example, which will be expanded later as a case study, is the abandoned zinc and copper mining operations within the city of Kabwe, Zambia, termed “The world's most toxic town” (Guardian, 2017). A victim of colonial mining, conducted with minimum concern of worker or population safety and maximum concern of profits, Kabwe has been left littered with mine tailings and waste dumps with concentrations of lead and other metals at toxic levels, resulting in profound lead poisoning epidemic in the city (Figure 7; Bose‐O'Reilly et al., 2017). Additionally, the tailings are so rich in lead that they are often secondarily mined by residents, with materials melted down in backyards and near homes for resale. Several active programs are underway in Kabwe to identify the most contaminated properties and mitigate them (Case Study 3), but the scale of the problem and the poor infrastructure for maintaining mitigation efforts (i.e., access to continuous and affordable irrigation to maintain grass groundcover) can limit effectiveness of these projects.

**Case Study 3 Measuring Pollution From Space**

**Figure 8:**
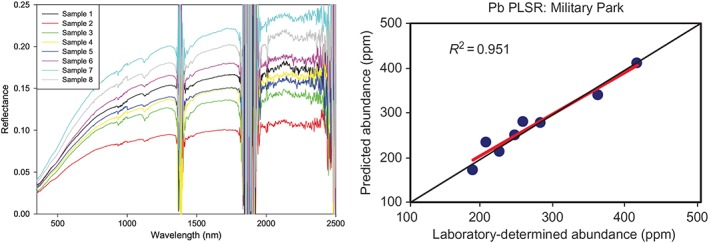
(left) Reflectance of soil samples, from a transect of near‐road (high lead) to far‐road (low lead) samples (sample 1 to sample 8), with reflectance measured outdoor and the noise at 1,400 and 1,900 nm is due to atmospheric water, (right) laboratory‐determined abundance of lead plotted against the abundance of lead predicted by a partial least squares regression model using 880 and 1,300 nm bands (from Pandit et al., 2010).

Planetary geologists have used the reflectance spectra from the Moon to interpret distribution of minerals and elements on surface soils (e.g., Li, 2006). Reflectance spectroscopy is a powerful tool as it provides interpretative resolution at the scale of the sensors used. The interactions of incoming solar radiation and minerals at the planetary surface can result in distinctive patterns of reflectance spectra that are correlated to mineralogic properties of the reflecting surface. The presence of water, organic matter, and photosynthesis‐active biological material significantly impacts the emission spectra—indeed, this characteristic is typically exploited to measure exactly these components on Earth. This approach shows extreme promise for mapping the concentration of metals in surface soils ((Figure 8); Pandit et al., 2010), particularly when hyperspectral detectors are used, which separate reflected light into narrow wavelength bands across a broad wavelength range. Although used for mining exploration (e.g., Bishop et al., 2011), these reflectance techniques have not widely been used to detect the distribution of pollution‐sourced metals and can be particularly important as a first‐level surveillance tool to identify hot spots for further focus. In a pilot effort in Kabwe, Zambia, a team of researchers is collecting and analyzing surface soil samples (top 1 mm) for the geochemical composition and the hyperspectral signatures, in an attempt to test current algorithms and prepare a predictive map for a subsequent drone‐based survey to validate this approach (Figure 8). It has the advantage of covering a broad area very quickly and for providing a very small spatial footprint of detection—on the order of <0.1 m. This technique, if validated, can be used to map broader areas of Kabwe and several other mining‐impacted cities in Zambia and Mozambique, capitalizing on the extended dry season that occurs in these areas that minimize interference from water and vegetation. This technique is not likely useful in all situations (i.e., tropical settings with significant vegetation cover and high soil moisture), but it can provide another sensing tool for 21st century identification of pollution hazards, with potential applications for satellite‐based hyperspectral tools.

## 4 Air Pollution and Health

### 4.1 History

Humans have profoundly altered the composition of the Earth's air throughout the twentieth and 21st centuries. Any time fuel is burned, it produces products of incomplete combustion, including air pollution, a mixture of gaseous, liquid, and solid compounds that damage the environment and human health. While air pollution is often viewed as an unfortunate byproduct of the Industrial Revolution, effects of emissions from coal burning were documented in cities all the way back to 375 BC Rome and likely before. It was not until the Belgium Meuse Valley (1930), the Donora Pennsylvania (1948), and the London (1952) smog disasters that air pollution really gained public and policy attention. Subsequent to these infamous events and their large associated death and morbidity tolls, domestic coal burning was prohibited in many parts of the world, and a wide industrial restructuring took place. Together with a gradual emergence of various clean air acts, environmental acts, and pollution control technologies, air quality improvements were realized across many regions and levels of several key pollutants declined considerably (Briggs et al., 1997). Since then, the story of air quality has evolved from a focus on local disasters and air quality management in the U.S. and Europe, to rapid increases in pollution levels in Asia and Africa, understanding of regional air pollution influences beyond national boundaries, and, more recently, recognition of interconnections between air quality and climate change. In present urban areas, the principal source of outdoor air pollution has also changed from coal‐based sources to road traffic, at least in developed nations.

### 4.2 Health Impacts

The health effects of air pollution are now well known and have been studied, confirmed, reanalyzed, and confirmed again through decades of environmental epidemiology and other types of health studies, including human exposure studies, animal studies, and cellular toxicology. Landmark studies that advanced the field and our understanding of the wide range of health effects associated with air pollution notably include the Harvard Six Cities study (Dockery et al., 1993) and the American Cancer Society Cancer Prevention Study (Krewski et al., 2005), both of which have been independently reanalyzed and further extended in numerous following publications. While these pioneering studies primarily focused on mortality, there is now emerging evidence that links air pollution to a wide spectrum of diseases including childhood asthma, congenital anomalies, dementia, low birth weight and preterm birth, lung cancer, obesity and diabetes, among others (Sanchez, 2020).

Based on this body of evidence, which continues to rapidly evolve, the World Health Organization (WHO), the European Commission and the U.S. Environmental Protection Agency (U.S. EPA), and other institutions around the world have developed causality determinations linking individual air pollutants and air pollution sources with health outcomes. These institutions also set ambient air quality guidelines (in the case of WHO) and regulations (in the case of U.S. EPA and the European Commission) at levels intended to be protective of public health. However, more recent evidence across a variety of health outcomes shows that adverse health effects are associated with air pollution levels well below current guidelines and regulatory standards, suggesting that these values do not reflect the latest evidence put forward by several epidemiological studies and that the science is outpacing legislation. Many studies have hinted at or suggested that air quality guideline values need revision, and, in fact, the WHO is currently in the midst of such revision to account for the most recent evidence linking air pollution to health effects.

### 4.3 Air Quality Trends

**Figure 9:**
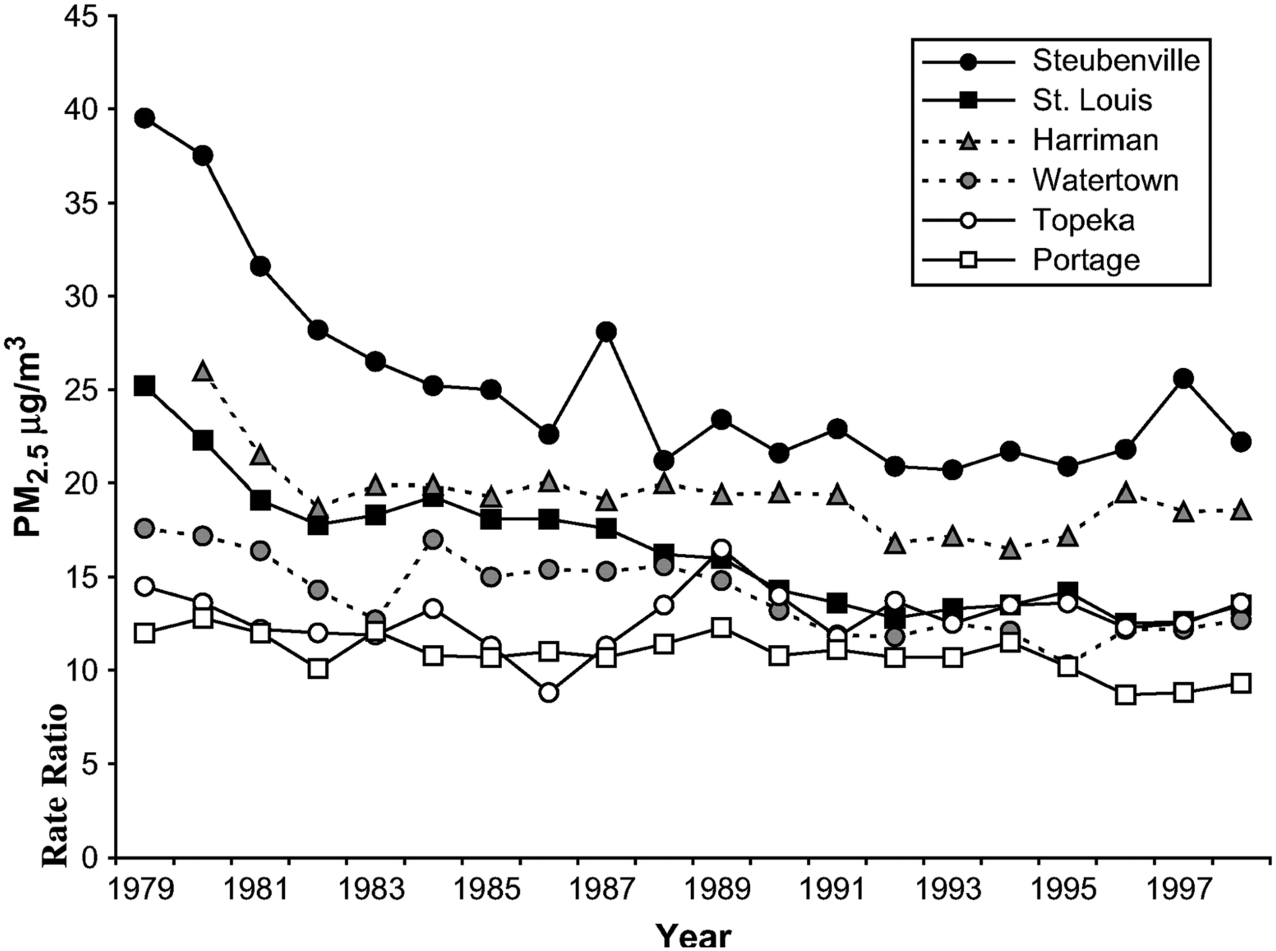
Annual average concentrations of PM_2.5_ in the Harvard Six Cities Study. Monitoring data for available years 1980–1988 and PM_2.5_ estimated from Aerometric Information Retrieval System and extinction data for years where Six Cities data were not available (from Laden et al., 2006).

Overall, trends in air pollution levels are mixed globally. While the air in developed nations has generally gotten cleaner even as economic conditions and productivity have grown, air pollution in developing nations continues to rise at an alarming rate (World Health Organization, 2016). Also, importantly, trends in air pollution and human exposures can significantly vary within cities and at the local scale. In general, air pollution, and therefore its associated adverse health effects, tend to be higher and more concentrated in lower socioeconomic locales and ethnic minority communities (Grineski & Collins, 2018; Morello‐Frosch et al., 2001; Mueller et al., 2018), despite some heterogeneity when observing in large metropolitan areas like New York (Hajat et al., 2013). In the U.S., for example, reductions in fine particulate matter emissions from U.S. manufacturing fell by about two thirds, even as real output from U.S. manufacturing grew substantially (Shapiro & Walker, 2019). Similarly, analysis by the U.S. EPA annual report: Our Nation's Air shows that concentrations of air pollutants have dropped significantly since 1990; for example, annual nitrogen dioxide (NO_2_) levels dropped by 56% and PM_2.5_ by 41% (U.S. Environmental Protection Agency, 2018). A recent nationwide analysis showed that NO_2_ levels across the contiguous U.S. decreased by 33% on average in the period between 2000 and 2010 (Alotaibi et al., 2019). However, the racial disparity between whites and nonwhites persisted in the same time period (Clark et al., 2017), indicating that although air quality is improving in the U.S., environmental justice is not (Figure 9).

### 4.4 Challenges to and Opportunities for Future Improvements

Ensuring healthy air quality for the global population throughout the 21st century could be challenged by anticipated global environmental change, large‐scale social and demographic shifts, and economic and technological developments. The overall air quality improvements in some regions over the last several decades have been largely achieved through emission control and abatement technologies, such as catalytic converters and diesel particulate filters on vehicles and scrubbers on power plants, in addition to productivity growth and movement of polluting industries, such as steel and cement, to places like China. Emissions, however, have been increasing in other parts of the world—notably in China throughout the 2000s and 2010s, and now in India. Large‐scale changes in the global environment, demographics, and economic and technological development in the future could affect population exposure to air pollution in different ways, and the pollution control measures relied on historically may not be enough in the future. Further, the emerging evidence showing consistent adverse health effects at low levels of air pollution is concerning and has great implications to risk assessment and policy decisions, as it indicates that health effects are still occurring at the lower levels of air pollution achieved in countries where air quality levels have improved over the last decades.

Effects of global climate change on air quality are increasingly studied and recognized as having a potentially profound impact on global public health. Climate change can affect ambient air pollution levels by changing photochemical rates of secondary pollutant formation and by increasing “natural” emissions (e.g., from soil dust and wildfire smoke). For example, climate change has been shown to increase ozone air pollution in the United States, particularly throughout the Midwestern states (Fann et al., 2015; USGCRP, 2016). Effects of climate change on particulate matter pollution are less well understood, and results of studies focusing on this topic are somewhat mixed due to multiple poorly understood and potentially competing effects occurring simultaneously. However, recent research indicates that climate‐driven meteorological changes can have strong effects on “natural” emissions. In the U.S., for example, climatic changes can lead to greater ambient soil dust concentrations in the Southwestern states (Achakulwisut et al., 2018; Achakulwisut et al., 2019) and increased smoke PM_2.5_ from wildfires throughout the country (Ford et al., 2018). While the net effect of climate change on air quality in different locations remains uncertain, the current evidence suggests potentially large impacts that are clearly deserving of further study.

Social and demographic changes may also influence air pollution emissions and impacts on public health throughout the 21st century, as has been shown to be the case over the last several decades (Cohen et al., 2017; Health Effects Institute and Institute for Health Metrics and Evaluation, 2019). These social and demographic changes include population aging, epidemiological shifts from infectious to noncommunicable diseases, and rapid urbanization, particularly in Asian and African countries. Potential future worsening of air quality throughout Africa and Asia is particularly concerning given that nearly all population growth projected through 2050 is expected to occur in African and Asian cities (United Nations, 2014). Health equity issues may also be exacerbated as wealthy nations in North America and Europe continue the trend of outsourcing manufacturing and associated pollution to other locations (Moran & Kanemoto, 2016; Zhang et al., 2017). In many cases, these countries have less stringent environmental regulations, resulting in higher emission factors per unit energy consumed. Populations in these locations often also have poor access to healthcare, medication, and other health systems, in addition to lower socioeconomic status and potentially poor diets and additional adverse coexposures, making them more vulnerable to health effects from air pollution. In total, population aging and urbanization combined with continued economic globalization may exacerbate the health impacts of air pollution, and inequities, over the coming decades.

**Figure 10:**
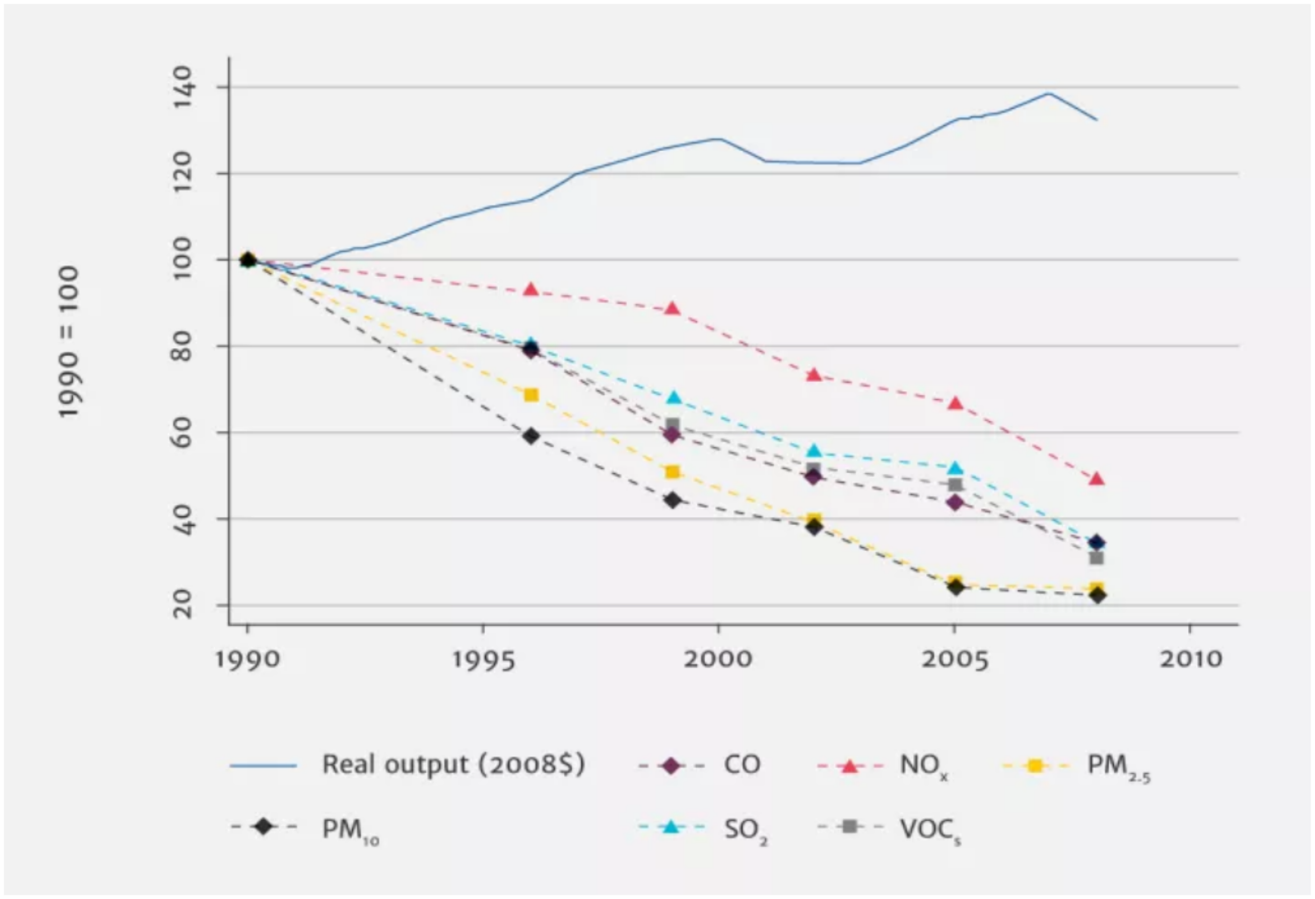
Trends in manufacturing pollution emissions and real output in the U.S. (from Shapiro & Walker, 2019).

Technological developments could also be game changers. The last few years have brought rapid declines in prices of renewable energy like solar and wind and shifts of energy production away from highly polluting coal (Watts et al., 2018). In the transportation sector, consumers are turning away from diesels in Europe in the wake of emissions cheating scandals, and hybrid electric vehicles are now cheaper than diesel vehicles in some parts of the world (Diaz et al., 2017). It remains unclear what large‐scale electrification of vehicle fleets, autonomous vehicles, and ride sharing will mean for air quality and public health, and only few studies have examined these issues. For example, if pervasive ride sharing displaces public transportation ridership or active commuting by bicycle and walking, transportation emissions may increase and physical activity could decrease. Vehicle electrification could improve air quality in cities but has the potential to increase pollution closer to electricity generating units where rural, nonurban car users might reside. Decarbonization of electricity generation as the price of renewables and natural gas drop makes vehicle fleet electrification increasingly attractive, at least from an air quality standpoint. Nonetheless, electricity generation is yet unclean in many parts of the world, and the relative importance of nonexhaust vehicle emissions, which may increase with fleet electrification due to increasing vehicle weights (Timmers & Achten, 2016), is often understudied. These technological changes may result in substantial air quality changes, and their influences on both combustion and noncombustion air pollution remain unknown (Figure 10).

**Figure 11:**
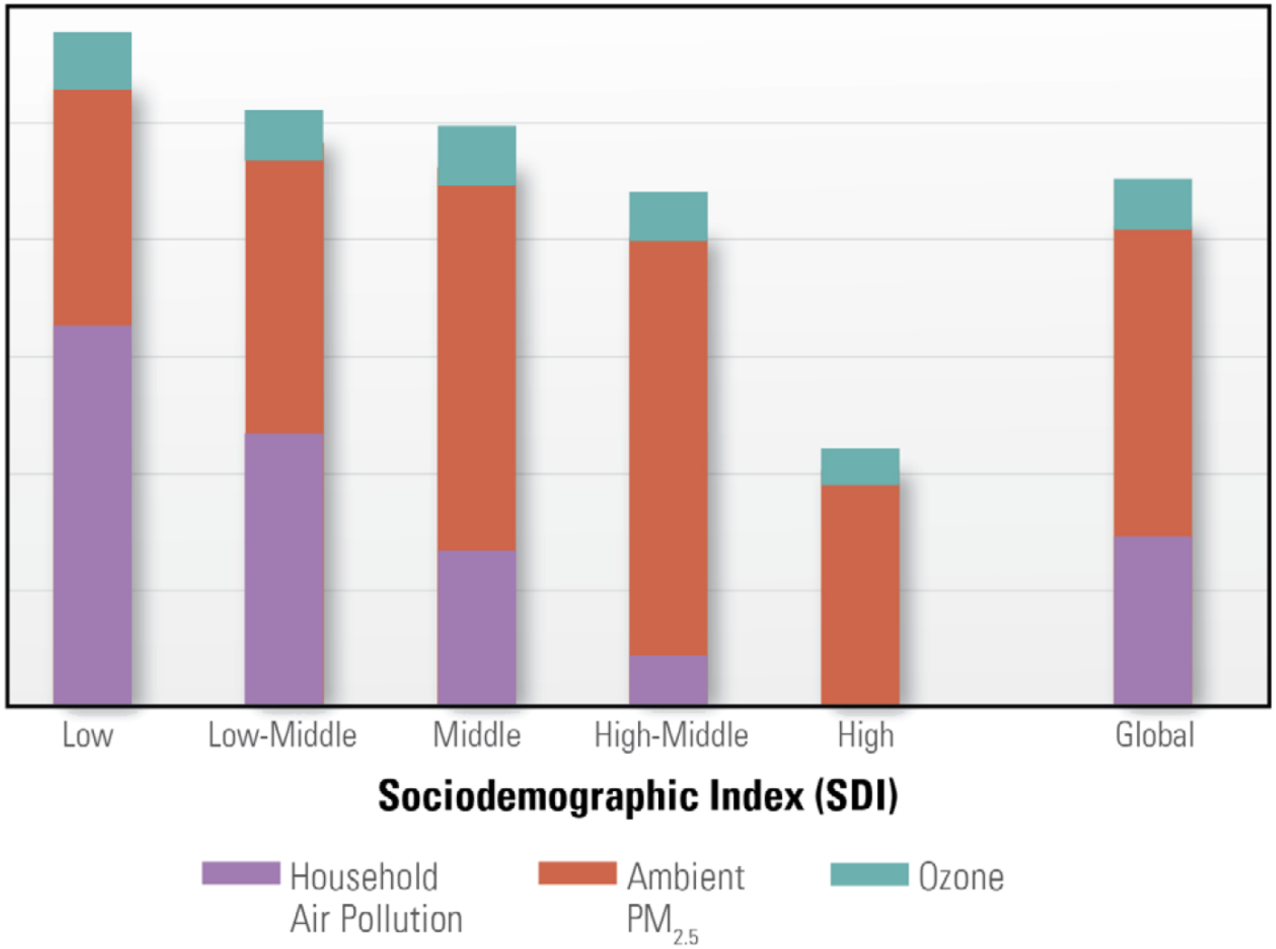
Comparison of percentages of deaths attributable to household air pollution, ambient PM_2.5_, and ozone by sociodemographic index (from Health Effects Institute and Institute for Health Metrics and Evaluation, 2019).

Economic shifts may also affect levels of exposure to both household air pollution and ambient air pollution. Economic growth and governments implementing large‐scale programs to electrify rural households and deliver cleaner fuels (e.g., LPG) and stoves are reducing the number of households that burn solid fuels inside or near their homes for cooking, heating, and lighting (Health Effects Institute and Institute for Health Metrics and Evaluation, 2019). Economic development may have the opposite effect on ambient PM_2.5_ levels—previous analyses indicate that as economies transition from low income to low‐middle and middle income, ambient PM_2.5_ and associated disease burdens rise (Figure 11; World Bank Group and Institute for Health Metrics and Evaluation, 2016). It will therefore be important to continue the transition away from household use of solid fuels and simultaneously accelerate adoption of world‐class emission standards and national ambient air quality standards to ensure that both household and ambient air quality improve for people around the world.

Major intergovernmental institutions have turned their attention to air pollution in recent years, even though it is not a major goal or target of the United Nations Sustainable Development Goals. The increased attention was sparked in part by recent “airpocalypse” events in China and India and the Global Burden of Disease studies that place air pollution as the leading environmental health risk factor globally, and the fifth leading health risk factor overall, resulting in millions of premature deaths worldwide each year. The Climate and Clean Air Coalition to Reduce Short Lived Climate Pollutants launched in 2012 and is aimed at mitigating both climate change and air pollution. In 2014 and 2015, the World Health Assembly and the United Nations Environment Programme, two high‐level platforms for making decisions on global health and the environment, passed resolutions to address air pollution. Other intergovernmental institutions, such as the Convention on Long‐Range Transboundary Air Pollution, have focused on air pollution many years earlier. Nongovernmental organizations are also taking action. One example is the C40 cities network (C40 Cities, 2019), which has traditionally focused on greenhouse gas mitigation and is currently expanding its focus on air quality management and working toward achieving climate, air quality, and health cobenefits in cities worldwide.

### 4.5 Air Pollution in the 21st Century

While the efforts described above have the potential to be transformative, more work needs to be done to promote awareness of air pollution and its public health consequences among the general public and decision‐makers at all levels—city, state, national, and intergovernmental. A key challenge that could hinder success in bringing down air pollution levels globally is the lack of funding. Air pollution has not received the same degree of investment as other global health risk factors, such as malaria and AIDS. In addition, many decisions affecting air quality are made at the local level, yet local jurisdictions often lack funding to address the issue to the degree that is necessary, given many other competing needs. Another key challenge is fragmented decision‐making at nearly all governance levels. Separate national or municipal administration departments address energy, transportation, air quality, and health, though these topics are highly interrelated and often interdependent.

Given what is currently known about the health risks from air pollution and its sources, what more needs to be done to fill knowledge gaps and track progress toward a goal of universal access to clean air? Open questions that warrant further investigation include a better understanding of mortality and morbidity impacts at low and high air pollution concentrations, which are two ends that are becoming increasingly relevant in developed nations as their air becomes cleaner, and in developing nations as their air pollution levels are at unprecedented peaks. Further, several studies have identified negative health effects of pollutants such as ammonia (a byproduct of Selective Catalytic Reduction technology and a contributor to the formation of secondary fine particles and ultrafine particles), black carbon, nonexhaust PM components, and ultrafine particles. In recent years, studies have shown that these pollutants are abundant on the local scale due to traffic emissions and that their toxicity might be heightened due to factors such as size, high concentrations (and numbers in the case of ultrafine particles), high surface areas, and toxic chemistry. The levels of these pollutants, however, are as yet unregulated in ambient air and are not routinely measured. More recent research has focused on NO_2_ as a relatively easy to measure marker of traffic, especially in urban areas, yet the putative agents especially in the traffic‐related air pollution mixtures remain largely unknown. Researching a wider range of air pollutants' effects, beyond PM_2.5_ and NO_2_, and specifically looking at subsets of particulate matter and their chemistry, might advance the science further. Another contemporary issue that warrants further attention is the relative importance of nonregulated, nontailpipe emissions in the burden of disease. These emissions are likely to increase, both in the developed and developing world with the expected widespread introduction of electric vehicles. Surprisingly, few studies have addressed the health effects of nontailpipe emissions, which again relates to the need for better PM speciation and analysis.

Further research and practice gaps remain. Historically, air pollution surveillance has relied on costly and resource‐intensive ground‐based monitors. We are now seeing rapid development of other types of technologies that are playing increasingly important roles in measuring air pollution concentrations. For example, satellite remote sensing of aerosol optical depth and trace gases have advanced considerably over the last decade and have been used to derive ground‐level estimates of PM_2.5_, NO_2_, and other pollutant concentrations globally (Duncan et al., 2014; Larkin et al., 2017; Shaddick et al., 2018; van Donkelaar et al., 2016). Rapid proliferation of low‐cost sensing technologies on the market, while still challenged by quality and durability issues, is likely to be increasingly used in citizen science contexts and may serve a role in air quality surveillance, health studies, and public awareness (more on this in the Case Studies section) particularly as the technology advances (U.S. Environmental Protection Agency, 2014). Pilot studies using air quality monitors on vehicles have also shown some promise in highlighting how air pollution levels differ between neighborhoods within cities (Alexeeff et al., 2018; Apte et al., 2017). Important limitations to using these new technologies remain, including challenges with achieving a high enough degree of accuracy and precision. The lack of ground‐based monitors in most of the world also makes it difficult to narrow uncertainties of concentration estimates from remote sensing and other technologies, as there are no observations to compare against. Advancing air quality surveillance globally should therefore remain a top priority for the global community in the coming years.

**Case Study 4 Sensors Everywhere**

**Figure 12:**
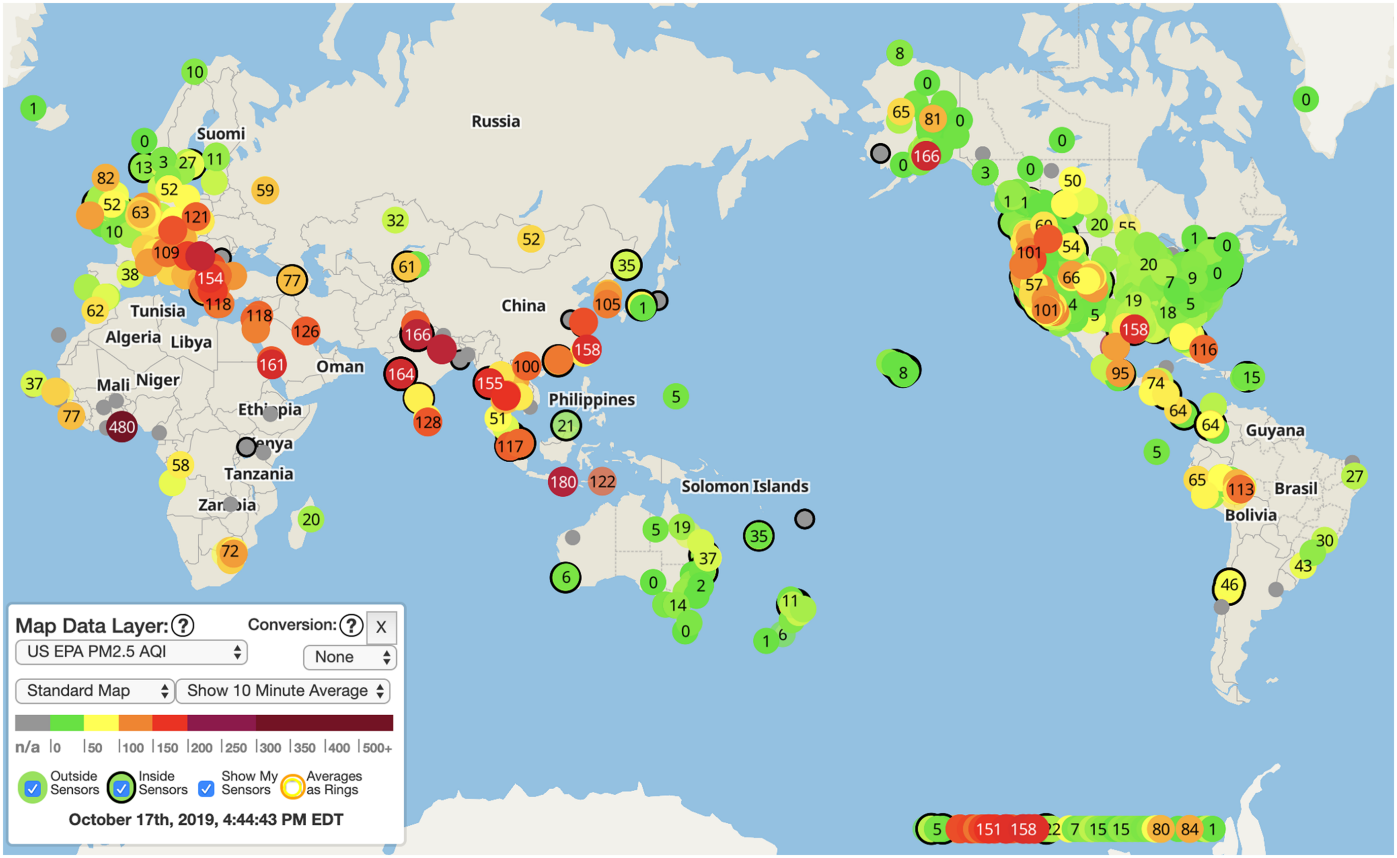
Screenshot of PurpleAir global map, showing the U.S. EPA PM_2.5_ Air Quality Index.

The explosion of wearable devices has brought a host of data to the personal level, whether it be fitness or health information via watches or air quality warnings via smartphones. Wearable systems have also been used to measure pollution exposure, including vest‐based systems for urban bikers in New York City to measure pollution exposure loads during bike commutes (Chew et al., 2019) and silicone wristbands used to measure exposure to a variety of human‐produced chemicals (e.g., Wang et al., 2019). Beyond wearables, a range of small‐scale environmental monitoring devices are now available and have been effectively deployed to measure air quality (mostly fine particulate matter PM_2.5_) at the local level. This has proven crucial in many environments where regulatory air monitors are sparse or even nonexistent and where hyperlocal measures are more important than regional measures of air quality. An example of a commercial system with wide application is PurpleAir, which has a detailed yet still approachable web presence to examine maps of air quality across the globe, updated every 60–90 s (Figure 12).

Research teams have used these networks to identify local risks to health, with the potential to offset the relative inaccuracy (compared to regulatory monitoring systems) with sheer number of sensors and with the ground‐level data that these sensors provide. An example of such an application is from the Santa Rosa, California fire of October 2017 (Gupta et al., 2018), where researchers used the PurpleAir network and compared results to the few scattered regulator monitors and with satellite‐based analysis of Atmospheric Optical Density (AOD) of PM_2.5_. This nested approach has the advantage of utilizing each technique to its maximum potential, with (1) PurpleAir monitors being of lower accuracy and precision as regulatory monitors for PM_2.5_ but capable of measuring this particulate at the personal level and on rapid timescales; (2) regulatory monitors being very widely spaced with placement specifically designed to capture regional air quality, but with data of high quality; and (3) satellite‐based sensors measuring the total atmospheric load of PM_2.5_ rather than just ground‐level data but having a broad coverage and including other meta‐data (e.g., IR to capture locations of active fires). This combination can bring disaster response from the level of space to backyards and significantly improves immediate response to protect public health.

There are emerging effects of air pollution that can substantially contribute to burden of disease analyses and provide a more complete picture of the true burden across a spectrum of outcomes beyond mortality, such as pregnancy complications and adverse birth outcomes, effects on fetal growth and birth defects, human reproduction, and neurotoxicity. The effects of air pollution are also not proportionally distributed despite the tendency in research (partly due to data availability and statistical power concerns) to estimate overall risk estimates in the total population rather than subanalyzing susceptible subpopulations. Extrinsic and intrinsic effect modifiers that have been identified in air pollution epidemiology, but that have not been systematically studied, include socioeconomic status, nutrition, stress, exposure to violence, coexposures (for example, noise, heat, and contamination), ethnicity, age, sex, and genetics. A better understanding of these factors, and a more specific exposure‐response function estimation in subpopulations, can help advance burden of disease assessment methods and better steer limited mitigation resources. Finally, there is a general consensus that the benefits of regulations and pollution abatement solutions outweigh the costs of implementation, especially when considering cobenefits beyond human health such as climate change mitigation. However, the benefits of solutions and regulations are very policy and context specific. Additional analysis, especially tracking the full‐chain between air pollution sources and their ultimate health impacts, can shed light on the results of specific measures in specific locations and populations and may strengthen the case for action.

Additional public health surveillance is needed to track how each emission sector is contributing to the air pollution disease burden globally, nationally, and in urban areas in response to changes in policies, technology, and population. Air pollution disease burden estimates should also be projected into the future to account for the environmental, technological, social, and economic shifts expected over the coming decades. Future projections can enable more informed decision‐making about how to mitigate the public health consequences of air pollution.

Often, decision‐making on air quality focuses on the lowest cost and fastest actions, evidenced by historical reliance in the U.S. and Europe on end‐of‐pipe emission controls, such as catalytic convertors and diesel particulate filters on vehicles and scrubbers on power plants. These technological controls have successfully reduced air pollution but do not reduce carbon dioxide or other greenhouse gas emissions and do not mitigate other societal damages linked to the same air pollution sources such as limited physical activity, urban heat, noise, road injuries, and fatalities. Instead, a more holistic approach beyond air pollution to consider other exposures and lifestyles is needed. Considering these other effects of air pollution sources can better frame linkages with human health and support analyses, policies, and strategies to holistically improve public health and avoid unintended consequences (Khreis et al., 2019). This more holistic approach points to the need for more fundamental changes to our energy systems and urban configurations. Fortunately, there are many ways to achieve multiple benefits for society simultaneously, including investing in active transportation, electrified public transportation, renewable energy for electricity generation, and building efficiency.

## 5 Democratizing the Science of Pollution Identification and Eradication

As illustrated in several of our Case Studies, and in our final Case Study 5, citizen science has a profound potential of democratizing science and placing facts and awareness in the hands of the very people who often benefit most by gaining agency from this information. In terms of pollution exposure research, one key responsibility is to effectively communicate how much a community should be concerned about the exposure, what they can do within their own capacity to minimize harm, and what role the community itself can and should play in alerting responsible parties and enforcing adequate mitigation. This can be particularly challenging among communities that have been disenfranchised from engagement through inequities in language, culture, race/ethnicity, and/or income compared to the majority population. One attempt to provide a consistent, available platform for citizen‐involved research and to communicate potential risks and provide interactive tools for assessment is provided by http://MapMyEnvironment.com (to disclose this is an app developed to collect data from and expand the breadth and depth of citizen science programs currently run by several of this paper's authors). At this point, this platform maps soil and dust geochemistry in a consistent manner across multiple programs and additionally can provide the capability of adding user‐added data on these media (Doyi et al., 2019). We envision the system accommodating multiple additional data layers, including water chemistry, more detailed information about indoor dust and the dust exposome (including allergens, flame‐retardant chemicals, antimicrobial resistance, to name a few), and any variety of other geolocated data that is not necessarily collected for environmental compliance.

Collaboration with communities involving site access and sampling demonstrates that science can actively respond to community demands for more evidence‐based knowledge and support their inventions. This might include interpreting citizen‐science collected data or via the provision of a service. Moreover, community engagement in scientific endeavors is an increasingly important aspect for researchers (e.g., Fryirs et al., 2019). Researchers are frequently required to demonstrate the societal benefit of their research, without which it is becoming increasingly difficult to gain access to the ever more competitive resources for research.

**Case Study 5 Science in the Hands of People**

The distribution of environmental contaminants of concern to human health is spatially often very heterogeneous. One example is the distribution of lead in surface soil. This heterogeneity has been shown, for instance, in Peruvian mining towns on the basis of rapid field surveys conducted with a handheld X‐ray fluorescence analyzer (Landes et al., 2019; van Geen et al., 2012).

**Figure 13:**
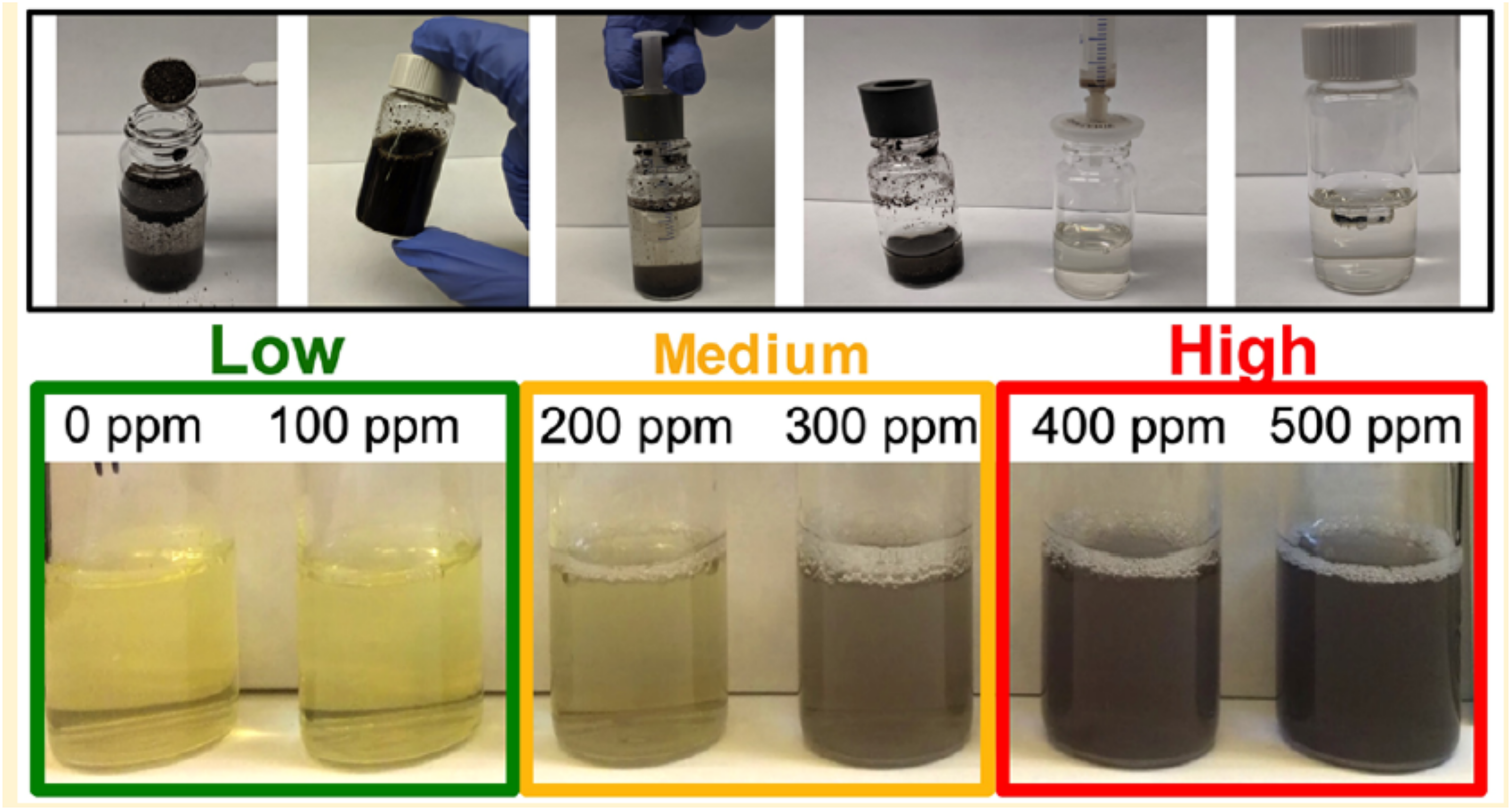
Application of a simple field procedure to screen soil for lead, using sodium rhodizonate as a color indicator for extractable lead concentration (from Landes, Inauen, et al., 2019).

Given the spatial variability of lead in soil, any area where children are likely to play and could ingest soil while doing so should in principle be tested. This is logistically and financially hard to imagine happening any time soon, especially in developing countries where priorities may lie elsewhere. A more feasible approach, therefore, is to involve parents in sampling and, preferably, testing their child's environment themselves. A field kit for screening soil for hazardous levels was recently developed with such an application in mind (Figure 13; Landes et al., 2019).

The soil kit combines a modification of the widely used method for extracting bioaccessible lead (Drexler & Brattin, 2007) with selective color detection using sodium rhodizonate. In order to achieve the necessary sensitivity, the soil/solution ratio of the extraction had to be increased tenfold relative to the original method. Inevitably, this reduced the fraction of lead extracted to about one third of that obtained with the original method but in a way that remained fairly constant across an environmentally relevant range of concentrations (Landes, Paltseva, et al., 2019).

**Figure 14:**
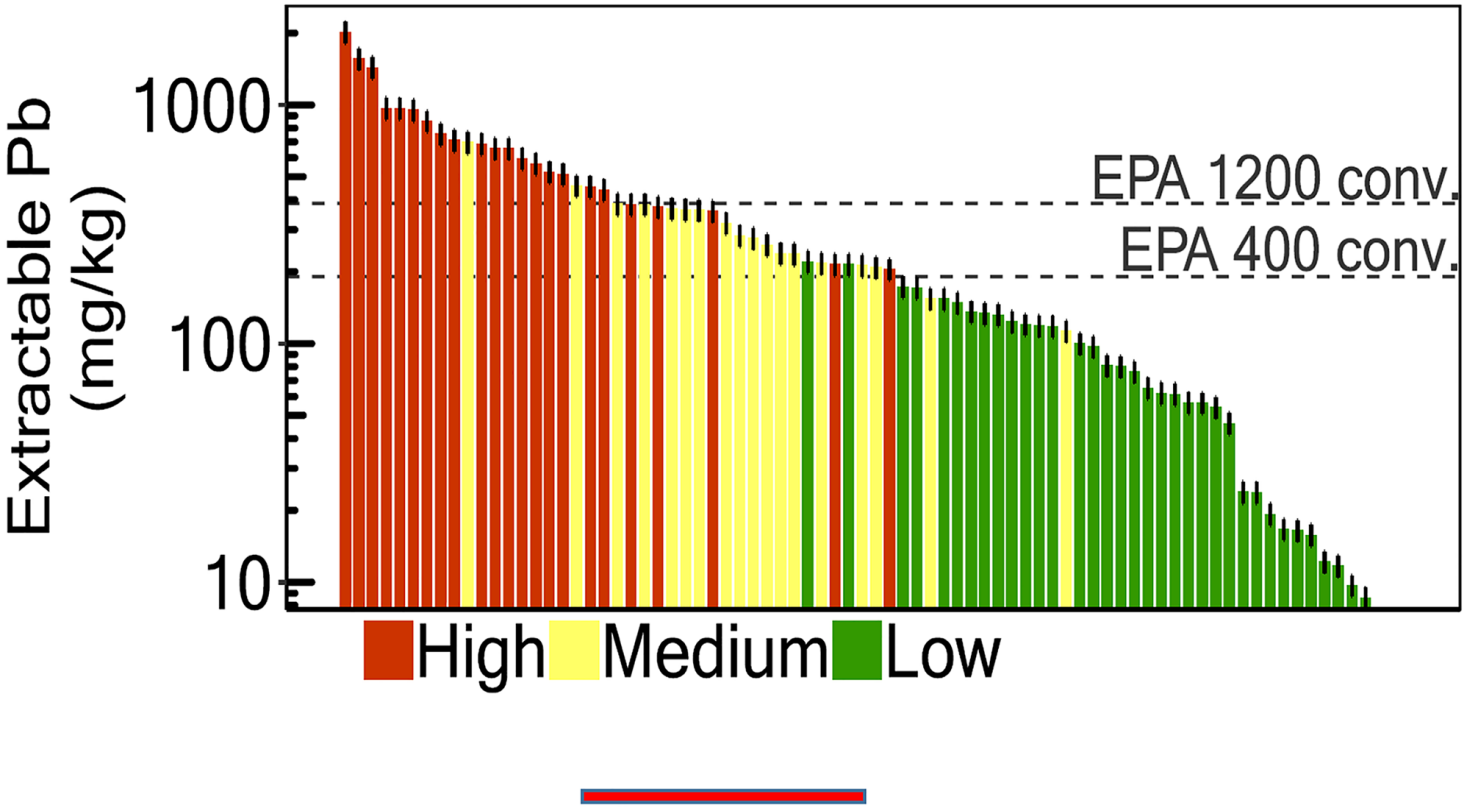
The kit results were recorded as high (clearly red or pink swab), medium (pink areas on paint itself), low (no red or pink anywhere) without knowing the lead content of the sample based on XRF (Landes, Inauen, et al., 2019).

Deployment of the kit has since been incorporated in the high school science curriculum of several high schools in Peru (Landes, Paltseva, et al., 2019). Under this project, all field data such as GPS coordinates and laboratory data are collected electronically using a widely used app (http://surveycto.com). For confirmation of the visual kit results, both the extracts and the soil samples collected by students are systematically analyzed by local support staff and the results communicated to the students and their parents. By and large, the visual and XRF measurements of lead concentrations in the extracts have been consistent. As a whole, the proportion of soil samples that exceed a threshold of 200–300 mg/kg extractable lead in soil is relatively well identified by citizen scientists using the colorimetric technique (Figure 14). Because of these limitations, this inexpensive technique should be considered a screening tool rather than a regulatory one, and high values clearly require confirmation by standard analysis. Regardless, the information is therefore well suited to students to learn about a locally relevant aspect of their environment and also for informing local authorities that follow‐up studies using more standard laboratory methods are needed.

**Figure 15:**
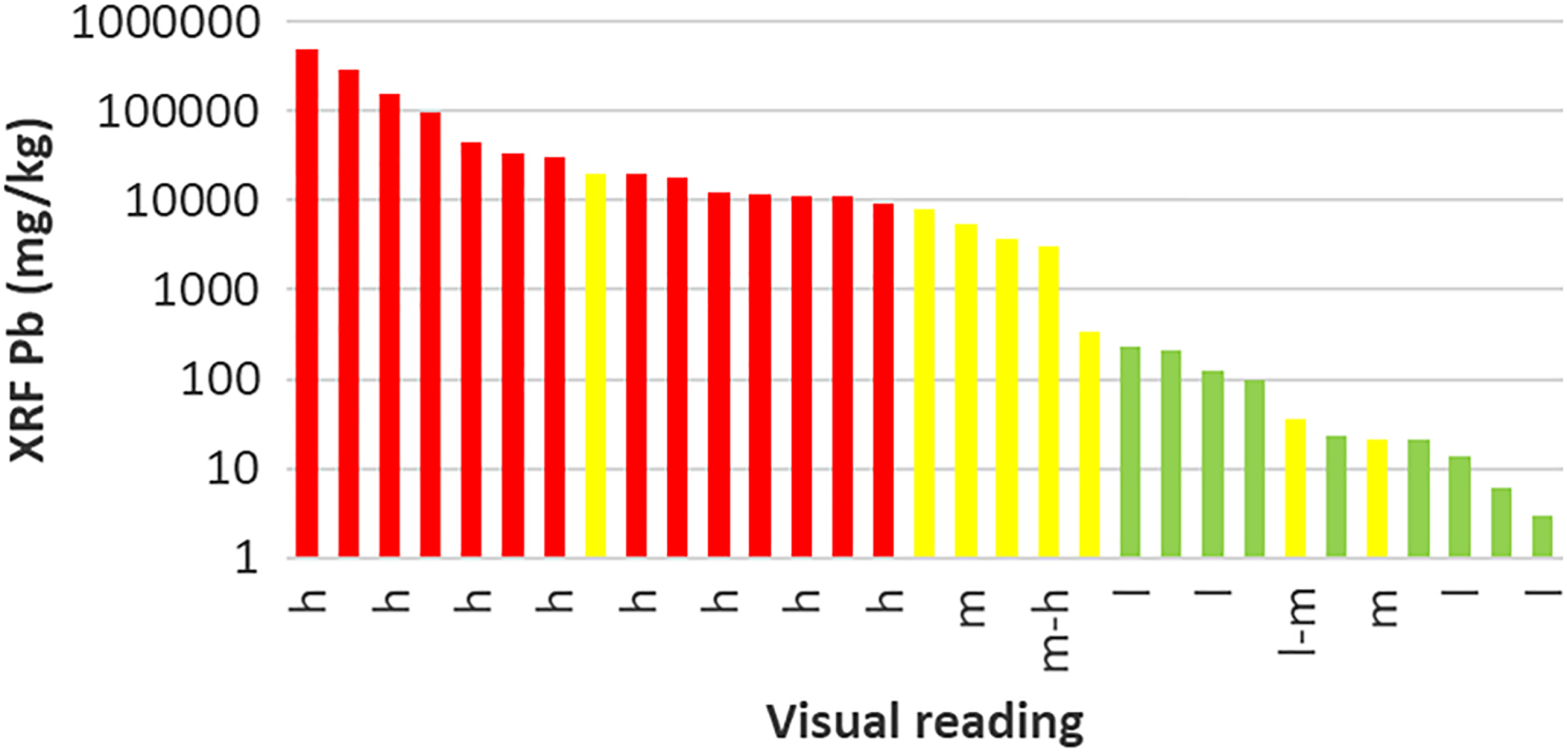
Comparison of Pb concentrations in paint measured by XRF with visual readings using the 3M kit: h = high, m = medium, l = low.

It is important to remain realistic about the scale of adoption of this new field kit. A recent study has been critical of the performance of a rhodizonate‐based 3M kit (Korfmacher & Dixon, 2007), but in our experience based on a comparison of kit results with XRF measurements, this kit reliably detects lead levels >1% (unpublished data based on paint samples from U.S. military bases provided by a team of reporters from Reuters: https://blogs.ei.columbia.edu/2018/08/21/lead-is-poisoning-children-on-u-s-military-bases/) (Figure 15).

## 6 Conclusions: New Paradigms for Reducing Pollution Burdens in the Next Century

This Centennial contribution has used several narrow examples to highlight the ways in which we have gained a greater understanding of the roles that the contamination of air, soil, and dust. It has also looked forward to where advances in this research should move to improve our global health outcomes in the face of multiple contamination inputs. It has not yet addressed several fundamental underpinnings of how we do our science in this area, and we argue that the current mechanisms for training of scientists, sharing data across fields and sectors, funding research, and translating research to action at the community level are woefully inadequate.

To address these shortcomings, we provided a series of Case Studies that exemplify novel emerging tools to bridge the science of pollution with the health of society. We suggest that the case studies of current work presage future developments in areas ranging from biosensing of contaminants, low‐cost testing kits and citizen science, and remote sensing approaches to detecting contaminants. This is in no way an exhaustive list but was aimed at providing some discrete examples of the bright future of identifying and addressing legacy pollution sources in soils and thus limiting the potential for soils and dust generated from them to harm people.

As our understanding of the complexity of the biogeochemistry and human exposure dynamics of pollutants has advanced, so too has our understanding of the complexity of individual human response to that exposure, as most research on pollution‐disease links has either been at the population level (so‐called ecological analyses) or focused on animal models. That gap in study design is wide and exemplifies the disciplinary gap between environmental sciences and human health sciences. Funding agencies are actively working to bridge that void by promoting highly interdisciplinary and convergent research. But this push also points to gaps in training and funding and research infrastructure, which will have to be filled in to make the headway needed. These current research funding priorities are designed to explore the question “What do we now know that we don't know in terms of pollution and health—the known unknowns?” We now know that the one chemical, one pathway, one human approach grossly underestimates the complexity of exposure science and disease and does not address at all the grave and unequal toll that pollution takes on global health.

## Conflict of Interest

The authors declare no conflicts of interest relevant to this study.
